# Extreme mortality and reproductive failure of common murres resulting from the northeast Pacific marine heatwave of 2014-2016

**DOI:** 10.1371/journal.pone.0226087

**Published:** 2020-01-15

**Authors:** John F. Piatt, Julia K. Parrish, Heather M. Renner, Sarah K. Schoen, Timothy T. Jones, Mayumi L. Arimitsu, Kathy J. Kuletz, Barbara Bodenstein, Marisol García-Reyes, Rebecca S. Duerr, Robin M. Corcoran, Robb S. A. Kaler, Gerard J. McChesney, Richard T. Golightly, Heather A. Coletti, Robert M. Suryan, Hillary K. Burgess, Jackie Lindsey, Kirsten Lindquist, Peter M. Warzybok, Jaime Jahncke, Jan Roletto, William J. Sydeman

**Affiliations:** 1 U.S. Geological Survey, Alaska Science Center, Anchorage, Alaska, United States of America; 2 University of Washington, School of Aquatic and Fishery Sciences, COASST, Seattle, Washington, United States of America; 3 U.S. Fish and Wildlife Service, Alaska Maritime National Wildlife Refuge, Homer, Alaska, United States of America; 4 U.S. Geological Survey, Alaska Science Center, Juneau, Alaska, United States of America; 5 U.S. Fish and Wildlife Service, Migratory Bird Management, Anchorage, Alaska, United States of America; 6 U.S. Geological Survey, National Wildlife Health Center, Madison, Wisconsin, United States of America; 7 Farallon Institute, Petaluma, California, United States of America; 8 International Bird Rescue, San Francisco Bay Center, Fairfield, California, United States of America; 9 U.S. Fish and Wildlife Service, Kodiak National Wildlife Refuge, Kodiak, Alaska, United States of America; 10 U.S. Fish and Wildlife Service, San Francisco Bay National Wildlife Refuge Complex, Fremont, California, United States of America; 11 Department of Wildlife, Humboldt State University, Arcata, California, United States of America; 12 National Park Service, Fairbanks, Alaska, United States of America; 13 NOAA Fisheries, Alaska Fisheries Science Center, Auk Bay Laboratories, Ted Stevens Marine Research Institute, Juneau, Alaska, United States of America; 14 Moss Landing Marine Laboratories, BeachCOMBERS, Moss Landing, California, United States of America; 15 NOAA Greater Farallones National Marine Sanctuary, Beach Watch, San Francisco, California, United States of America; 16 Point Blue Conservation Science, Petaluma, CA, United States of America; Hawaii Pacific University, UNITED STATES

## Abstract

About 62,000 dead or dying common murres (*Uria aalge*), the trophically dominant fish-eating seabird of the North Pacific, washed ashore between summer 2015 and spring 2016 on beaches from California to Alaska. Most birds were severely emaciated and, so far, no evidence for anything other than starvation was found to explain this mass mortality. Three-quarters of murres were found in the Gulf of Alaska and the remainder along the West Coast. Studies show that only a fraction of birds that die at sea typically wash ashore, and we estimate that total mortality approached 1 million birds. About two-thirds of murres killed were adults, a substantial blow to breeding populations. Additionally, 22 complete reproductive failures were observed at multiple colonies region-wide during (2015) and after (2016–2017) the mass mortality event. Die-offs and breeding failures occur sporadically in murres, but the magnitude, duration and spatial extent of this die-off, associated with multi-colony and multi-year reproductive failures, is unprecedented and astonishing. These events co-occurred with the most powerful marine heatwave on record that persisted through 2014–2016 and created an enormous volume of ocean water (the “Blob”) from California to Alaska with temperatures that exceeded average by 2–3 standard deviations. Other studies indicate that this prolonged heatwave reduced phytoplankton biomass and restructured zooplankton communities in favor of lower-calorie species, while it simultaneously increased metabolically driven food demands of ectothermic forage fish. In response, forage fish quality and quantity diminished. Similarly, large ectothermic groundfish were thought to have increased their demand for forage fish, resulting in greater top-predator demands for diminished forage fish resources. We hypothesize that these bottom-up and top-down forces created an “ectothermic vise” on forage species leading to their system-wide scarcity and resulting in mass mortality of murres and many other fish, bird and mammal species in the region during 2014–2017.

## Introduction

Marine heatwaves (hereafter “heatwaves”), defined as prolonged periods where ocean temperatures are much warmer than usual [1,2], have recently emerged as a major mode of ocean-climate variability that can significantly alter marine ecosystem structure, phenology and marine species distributions [3,4]. Heatwaves have become more prevalent and intense over the last century [4]. Under climate change projections of 2–3.5°C warming relative to pre-industrial levels, the expected intensity, frequency, spatial extent and duration of heatwaves by the end of the twenty-first century may well cause unprecedented and irreversible changes to marine ecosystem functionality and stability [5]. Here we examine impacts of a recent severe heatwave on marine ecosystems of the northeast Pacific and discuss some potential mechanisms by which extreme ocean heating has affected pelagic food-webs in this region.

During late 2013, a warm temperature anomaly developed in near-surface (upper ~100m) waters well offshore in the Gulf of Alaska (GOA) which grew to encompass a large area of the northeast Pacific Ocean [6]. Offshore sea surface temperatures (SSTs) during the winter of 2013–2014 exceeded 3 standard deviations in some areas and persisted over much of the central GOA through March 2015 [6]. While the offshore anomaly diminished somewhat through the summer of 2014, positive SST anomalies over the entire northeast Pacific re-intensified towards the end of 2014, moved into the coast [7] and persisted into the fall and winter of 2015–2016 [3]. Maximum temperature anomalies at times exceeded 3–6°C throughout the range of the heatwave from southern California [7] to the GOA [3], and extended to depths of ca. 50–200 m [8,9]. The period in which temperatures exceeded climatological thresholds for a sustained period (Aug 2014- July 2016) has been classified as a “severe” (Category III) heatwave [10]. At the time of publication, the spatial extent, magnitude and duration of this heatwave were the largest on record [4,6].

Some immediate biological effects of this unprecedented heatwave were equally extreme. For example, phytoplankton production in the central north Pacific waters was greatly reduced [11]; the largest harmful algal bloom in recorded history extended from California to the GOA in 2015 [12,13]; a massive die-off of planktivorous Cassin’s auklets (*Ptychoramphus aleuticus*) occurred from central California to British Columbia in the winter of 2014–2015 [14], a marked increase in mortality of pinnipeds was noted in southern California [15], and an unusually large die-off of baleen whales occurred in the GOA in 2015–2016 [16].

We report on another extreme biological impact of the 2014–2016 heatwave: The widespread die-off and chronic reproductive failure of a trophically dominant piscivorous marine bird, the common murre (*Uria aalge*), over much of their northeast Pacific distributional range from the southeast Bering Sea south to the California Current System (CCS). Murre die-offs occur irregularly in the Pacific and Atlantic oceans, often on wintering grounds, during stormy conditions, and under circumstances where food supplies are depleted or unavailable [17,18]. They have also been linked with warm water anomalies, such as the strong El Niño events in 1983, 1993, and 1998 [19–21].

Here we document the magnitude of the 2015–2016 murre die-off in terms of its spatial extent, duration, absolute numbers of dead or dying birds recovered, and relative magnitude of deposition on beaches relative to long-term baselines. We used data collected by systematic and repeated surveys for beached birds conducted by citizen science participants in the northeast Pacific [22], opportunistic surveys conducted in Alaska by government, university and private organizations, community reports, and records from bird rehabilitation centers. We also document concurrent reproductive failures of murres at multiple breeding colonies from Alaska to California. We hypothesize that the northeast Pacific heatwave was a source of simultaneous bottom-up and top-down forcing, and we discuss potential mechanisms for the disruption of food supplies that resulted in murre mortality and reproductive failure.

## Materials and methods

### Oceanography

We used sea surface temperature (SST) data from the Hadley Center Sea Ice and Sea Surface Temperature (HadISST) data set [23] to illustrate temperature perturbations related to the northeast Pacific heatwave. A time series of average SST anomalies (SSTa) for the GOA and the California Current System (CCS) were calculated for years 1870–2018. Areas that were analyzed separately included the GOA region: 50°N-61°N, 160°W-126°W; CCS northern region: 40°N-46°N, 126°W-124°W; CCS central region: 35°N-40°N, 126°W-120°W; and CCS southern region: 31°N-35°N, 120°W-117°W. The seasonal cycle was calculated by averaging all values of each month for the period 1870–2018. We subtracted the seasonal cycle (mean SST) from the SST to obtain the anomaly time series (SSTa). We estimated annual rates of SST change between years by calculating ΔSST = SSTYearN+1 –SST_YearN_ and then calculated a 5-year running average to obtain an annual index of SST change rate (°C year^-1^).

### Ethics statement

Specimens that were salvaged for necropsies and testing for disease, biotoxins, etc., were collected under U.S. Fish and Wildlife Service (USFWS) permit, i.e., the Migratory Bird Master Permit/Import-Export (MB025076-0) and State of Alaska Department of Fish and Game (ADF&G) Scientific Permit issued to the USFWS Regional Director. U.S. Geological Survey personnel operated under the same permits, as issued to the Alaska Biological Science Center USFWS (MB789758-2) and the ADF&G.

### Beach surveys

Effort-standardized surveys for beachcast marine birds at monthly or more frequent intervals were conducted at predetermined survey sites by participants of three beached bird citizen science programs: Beach Coastal Ocean Mammal/Bird Education and Research Surveys (BeachCOMBERS) [24], Beach Watch [25], and the Coastal Observation and Seabird Survey Team (COASST) [22] (Table 1). Standardization was achieved primarily by measuring the distance of beach transects (generally less than a few km) and calculating encounter rates as [birds found]/[km searched]. Other protocols designed to make comparisons among beaches, date and location more accurate usually included: walking the same area of beach each visit, surveying a strip centered on the most recent high-tide wrack line and adjacent zones, identifying species from field guides and photographs, marking carcasses to avoid duplicate counting, and searching during ebbing or low tides [22,24–27]. All three programs provide participants with extensive training in both survey protocol and carcass identification (which were verified photographically). Because BeachCOMBERS (34.04°-36.98°N) and Beach Watch (37.12°-38.97°N) programs occur only along the California coast, we assumed all unidentified murres were common murres. For COASST surveys conducted from northern California to northern Washington (39.13°-48.34°N) we made the same assumption. In the Gulf of Alaska (bounded from SE Alaska corner 54.75°N, -130.30°W, to northern Cook Inlet 61.31°N, -150.71°W, and west to Unimak Pass 54.29°N, -165.06°W), both common and thick-billed murres (*Uria lomvia*) were possible and species were identified based on bill morphology and facial plumage [28]. Less than 1% of all murres in the COASST dataset were identified as thick-billed murres, and most of those were in the Bering Sea (as far north as 68.80°N, -163°W, as far west as 52.30°N, 176.08°E). For simplicity, we assumed that unidentified murres were common murres.

**Table 1:** Standardized beached bird survey effort, number of murres reported on surveys, and murres reported by members of the public or delivered to rehabilitation centers between May 2015 and Apr 2016. Sources: COASST- Coastal Observation and Seabird Survey Team, DOI- Department of the Interior agencies, B.Watch- Beach Watch, COMBERS- BeachCOMBERS.

Location	Standardized beach surveys					Number of murres reported			
	Source	Start	#sites	#surv	#km tot	Surveys	Public	Rehab	TOTAL
Chukchi	COASST	2006	5	25	47	14		0	14
Aleutians	COASST	2006	7	34	39	10	200	0	210
Bering Sea	COASST	2006	9	110	114	23	300	0	323
Gulf of Alaska	COASST	2006	52	423	359	4289		0	4289
Gulf of Alaska	DOI	2015	114	164	381	20240	21435	552	42227
SE Alaska	COASST	2006	11	91	124	11	383		394
Salish Sea	COASST	1999	210	1771	2075	10			10
N Washington	COASST	1999	37	310	492	884		24	908
S Washington	COASST	1999	41	381	564	914		108	1022
N Oregon	COASST	2001	55	476	645	1249		332	1581
S Oregon	COASST	2001	34	232	345	353		3	356
N California	COASST	2006	47	403	428	357		52	409
C California	B.Watch	1994	40	975	1516	2927		680	3607
SC California	COMBERS	1994	46	403	1125	5071		1614	6685
TOTALS			708	5798	8253	36352	22318	3365	62035

All carcasses found were marked or removed to prevent recounting. For analysis of carcass deposition per effort (km walked), we divided program surveys into two categories: event (May 1, 2015 to April 30, 2016) and baseline (all surveys prior to May 2015). Because each program was created at different points in time (Table 1), and expanded at different rates, we calculated two baselines: (1) 2006–2015 across all programs (i.e. baseline period was invariant across locations); (2) program-specific start year through 2015. We only use the first baseline (2006–2015) for analyses in this paper, even though it meant discarding data, because it differed little from program-specific baselines (S1 Text) and it standardizes data for program comparisons.

Additional surveys were undertaken in Alaska, including 164 standardized [26], effort-controlled surveys conducted on 114 beaches by several Department of Interior agencies (U.S. Geological Survey [USGS], U.S. Fish and Wildlife Service, National Park Service) and opportunistic data collected by the public on beaches, inland or at sea (beach-walker, hiker, hunter, boater) or other wildlife biologists at 260 sites visited for other purposes. Public Opportunistic data occasionally included an estimate of beach survey effort (in linear km), but survey effort and carcass numbers were most often approximated. Some murres were also encountered inland and along lakeshores, and locations were attributed to a single point source.

### Specimen collections and necropsies

Specimens were collected during the study period (May 1, 2015 to April 30, 2016) and examined in three different efforts:

#### Rehabilitation centers

We contacted 72 bird rescue and rehabilitation (“rehab”) centers from southern California to Alaska (S1 Table) to obtain data on common murres recovered from beaches by the public and examined by veterinarians or other staff during 2015–2016 (S1 Fig). Of 66 that responded, 29 reported no common murre recoveries and 37 reported intakes totaling 3,365 murres. Of those, 2,868 birds were visually or manually examined for condition or diagnosis (e.g., sick, emaciated, dead on arrival, injured, oiled) at the time of intake, with ultimate disposition subsequently recorded (e.g., died, rehabilitated and released, etc.). Birds described variously as emaciated, thin, starving, skinny, underweight, or malnourished were simply categorized as emaciated. No birds were excluded from the tally of total birds encountered because: 1) there is always some background deposition of murres on beaches due to other mortality factors in addition to starvation, and so these should not be excluded as we compare 2015–2016 mortality rates against historical averages, and, 2) murres may be oiled or injured as they drift towards shore in coastal waters with heavy boat traffic and chronic oil pollution, 3) the diagnoses are not mutually exclusive. The majority (90%) of birds died prior to or soon after intake. The remaining 10% were released back into the wild, often without rehabilitation where facilities were limited, and their survival rate was likely low. Just under half of birds (and few from Alaska) were weighed on intake (n = 1,568) and aged (n = 1,298) based on morphometrics and/or plumage following Pyle et al. [29]. Subsets of each of these samples were created to delineate juvenile (HY) from older birds (AHY, includes adults, subadults) birds (S2 Table).

#### USGS National Wildlife Health Center (NWHC)

Common murre carcasses were collected from multiple coastline locations along the GOA (n = 89) and southern Bering Sea (n = 14) and shipped to the NWHC for diagnostic examination by American Veterinary Medical Association certified pathologists [30]. We sought out birds that were “fresh”, i.e., they had been dead less than a couple days to a week, all body parts were intact, and birds had not been scavenged or have exposed muscle or bone [27]. Measurements, ancillary laboratory testing and postmortem findings to support cause of death determination varied by individual specimens based on carcass and tissue postmortem quality (S2 Table). Weight (n = 90) and sex (n = 87) were recorded. Age class (n = 101) was determined from bursa of Fabricius, thymus and gonad development as: juvenile (HY), and subadult or adult (AHY). Samples were collected from the proventriculus or cloaca, if available, and analyzed for saxitoxin (n = 39) and domoic acid (n = 9) exposure at the National Oceanographic and Atmospheric Administration, Northwest Fisheries Science Center, Seattle, Washington, US using enzyme-linked immunosorbent assay (ELISA; [31]).

#### USGS Alaska Science Center

Fresh (see above) common murre carcasses (n = 117) collected off Alaskan beaches (n = 88) between 1 November 2015 and 11 April 2016 or provided by Alaska rehab centers (n = 29), were necropsied by agency biologists in Anchorage, Alaska (S2 Table). Not all characteristics were assessed on all birds, and subsets of data were collected for each parameter, including mass (n = 97), body condition, which we rank-scored by visually assessing amounts of pectoral muscle (n = 101) and subcutaneous fat (n = 102) [32], age (n = 36) and sex (n = 105) [33]. Birds were aged as hatch year (HY) if they had a bursa [34,35] and if gonads were not developed, if plumage (outermost underwing primary covert) were white-tipped, characteristic of HY/SY [29] and if culmen length was <40 mm [29]. AHY body mass was contrasted to 219 AHY specimens collected at seven GOA breeding colonies between May and September 1988–1999 by USGS (J. Piatt, unpubl.), as well as to carcasses (n = 116) recovered during a previous die-off in the GOA [20]. In these murres, any bird with AHY body mass approaching or falling below 650 g would be considered in Phase III starvation [36] and in immediate danger of dying.

### Murre breeding ecology

Reproductive success (*rs* = chicks fledged/egg laid) of common murres was obtained from 21 monitoring sites in our study area (13 in Alaska, 1 in Oregon, and 6 in California), including data collected by Alaska Maritime National Wildlife Refuge (NWR), Togiak NWR, Becharof NWR, USGS Alaska Science Center, Alaska Department Fish and Game, Institute for Seabird Research and Conservation, Oregon State University, Humboldt State University, Point Blue Conservation Science, San Francisco Bay NWR Complex and Farallon Islands NWR. Data collection consisted of systematic recordings (photographic or hand-drawn) of the status (i.e., presence of egg or chick) of a subset of nest sites within long-term plots [37]. Time series of reproductive success varied in length from 10 to 45 years among colonies. Standardized anomalies of breeding success were calculated for the entire time series of each colony, but anomalies were plotted using: a) only data collected after 1984 (owing to scarcity of data at most colonies in earlier years), and, b) using a mean *rs* for the baseline period 1996–2014; the period for which regular annual monitoring was initiated at 6 new index colonies in Alaska by Alaska Maritime NWR, and which has a near-complete time series at all but 2 of 11 long-term sites used in this study (Yaquina Head, Castle Rock N). For analysis, we consider the time series divided into event: 2015–2017, and pre-event baseline: 1996–2014.

Under a wide range of prey densities, average common murre *rs* is usually high and variance is low (e.g., [38,39]). At 11 colonies dispersed throughout their range in the northeast Pacific (n = 246 colony-years), mean *rs* (±SD) during 1972–2014 was 0.55 ±0.20 chicks fledged/egg laid. Thus, we defined a “reproductive failure” as *rs* that fell more than 2 SD below that mean (i.e. < 0.14), rounded down here to *rs* ≤ 0.10 chicks fledged/egg laid. “Complete reproductive failure” is defined as *rs* = 0, when no chicks whatsoever are produced.

### Data analysis

To illustrate spatial magnitude of the event, carcass count data of murres on both systematic and opportunistic beaches sampled, and records of live and moribund murres rescued from point locations were compiled for the event period (May 1, 2015 to Apr 30, 2016). Raw data were binned into 75x75 km cells for mapping; a scale big enough to aggregate higher resolution (km) beach surveys or point samples and prevent excessive overlap of adjacent abundance circles, but small enough to track coast and island geography (individual beach survey sample sites are plotted in S2 Fig). Not all shoreline was searched for birds during the die-off period, particularly in Alaska. Thus, the map underestimates the extent of the die-off in Alaska and may overemphasize the die-off in regions with a high density of survey sites on the coasts of California, Oregon and Washington (hereafter “West Coast”).

Deposition of carcasses on beaches is a measure that incorporates both detection and persistence and is proxied by carcass encounter rate. To examine the seasonal variation of relative carcass abundance within and among large geographic regions, raw beach survey data from citizen science programs (above) were standardized to carcasses encountered per linear km of coastline by survey [14] and averaged within geo-region by month (May 2015 to April 2016; Table 1). Relative magnitude of carcass encounter rate was calculated as 2015/2016 encounter rates divided by the baseline encounter rate, presented as a month-specific average across all baseline years (GOA: 2005–2014, Washington: 2001–2014, Oregon: 2001–2014, N. California: 2006–2014, N. Central California: 1994–2014, S. Central California: 1997–2014). Magnitude was also calculated using an equivalent baseline time period (2006–2014) for all geo-regions (S1 Text).

In order to determine whether carcass encounter rates in 2015/16 were significantly higher than previous years we calculated bootstrap 95% confidence intervals of mean encounter rate at the region and month-year scale. Each bootstrap estimate was calculated by drawing n samples (with replacement) of survey-specific encounter rate from the pool of available surveys for that month-year and region (Gulf of Alaska, Outer coast of Washington, Oregon, N California, C California and SC California–see Table 1), with n equal to the number of unique beaches surveyed in that month-year. A distribution of mean encounter rate was then generated by performing 1,000 bootstrap permutations, subsequently processed to obtain a 95% confidence interval specific to that month-year and region.

The average for each calendar month (i.e. the baseline) was then calculated by a second round of bootstrap resampling using the distributions generated in the previous step (S1 Text). Monthly encounter rates from May 2015 to April 2016 were then compared to the long-term baseline to identify whether they were significantly higher/lower than expected. We classified each month according to two significance criteria; (1) whether the encounter rate was significantly higher/lower than the long-term average (i.e. no overlap of corresponding 95% CI’s), (2) whether the encounter rate was significantly higher than any prior year for that calendar month (i.e. 2015/16 data was higher and had none overlapping 95% CI’s compared to all prior years) (S1 Text).

## Results

### Oceanography

**Fig 1:**
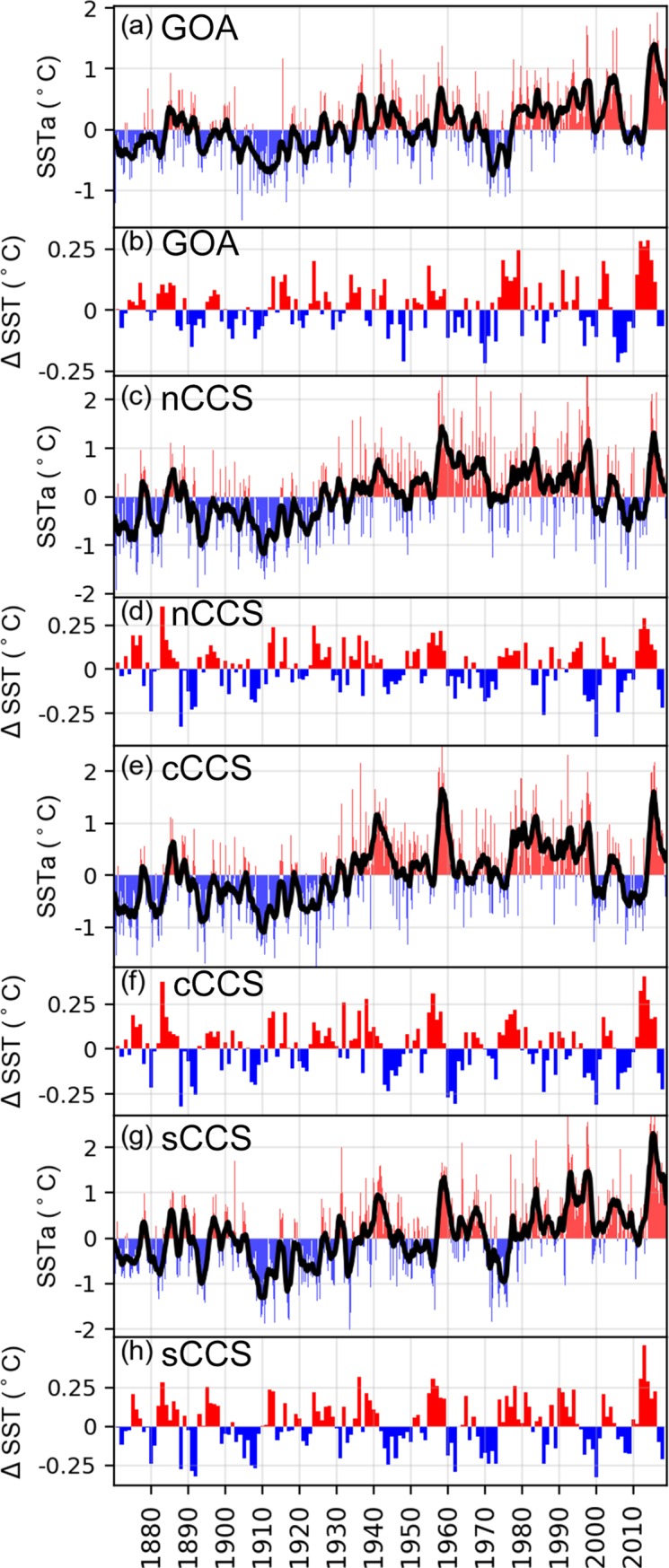
Average monthly time series (Jan 1870 to Dec 2018) of sea surface temperature anomalies (SSTa) in the (a) Gulf of Alaska (GOA); and in (c) northern (nCCS); (e) central (cCCS); and (g) southern (sCCS) waters of the California Current System (CCS). Solid black lines are 24-month running averages. Also presented are 5-year running averages of annual SST differences, an index of SST change rate, for the (b) GOA, and (d) nCCS, (f) cCCS and (h) sCCS. Average long-term SST values for each region are: GOA 8.1° C, nCCS 12.5° C, cCCS 13.9° C, sCCS 16.2° C.

The observed warming in the GOA from winter 2014 through winter 2016 was unprecedented in the period since instrumental record-keeping began (1870–2017; Fig 1A). The overall change in magnitude (Fig 1A), and rate of temperature change (Fig 1B), from the most recent cold anomaly (ca. 2007–2012) to the peak warm anomaly (2014–2016) exceeded any previous warming event in the GOA. While the magnitude of SSTa and rate of change in the northern CCS were notable, they were not unprecedented. In the central CCS, the magnitude of the SSTa was large but not unusual, whereas the high rate of warming was greater and more persistent than any time in the past. In the southern CCS (including most of California), the SSTa and rate of change were more extreme than even those in the GOA, and unprecedented in the ~150-year time series. Thus, while all areas were affected by the heatwave, and each developed strong temperature anomalies, it appears that both the heatwave magnitude and rate of warming were most extreme in the northern and southern reaches of its extent. It is noteworthy that while the “heatwave” did not reach the anomalous warm temperatures that defined it [1,10] until August of 2014, high rates of increase (>0.25 ΔSST) actually began much earlier, i.e., during 2012 in all areas.

### Die-off event description

**Fig 2:**
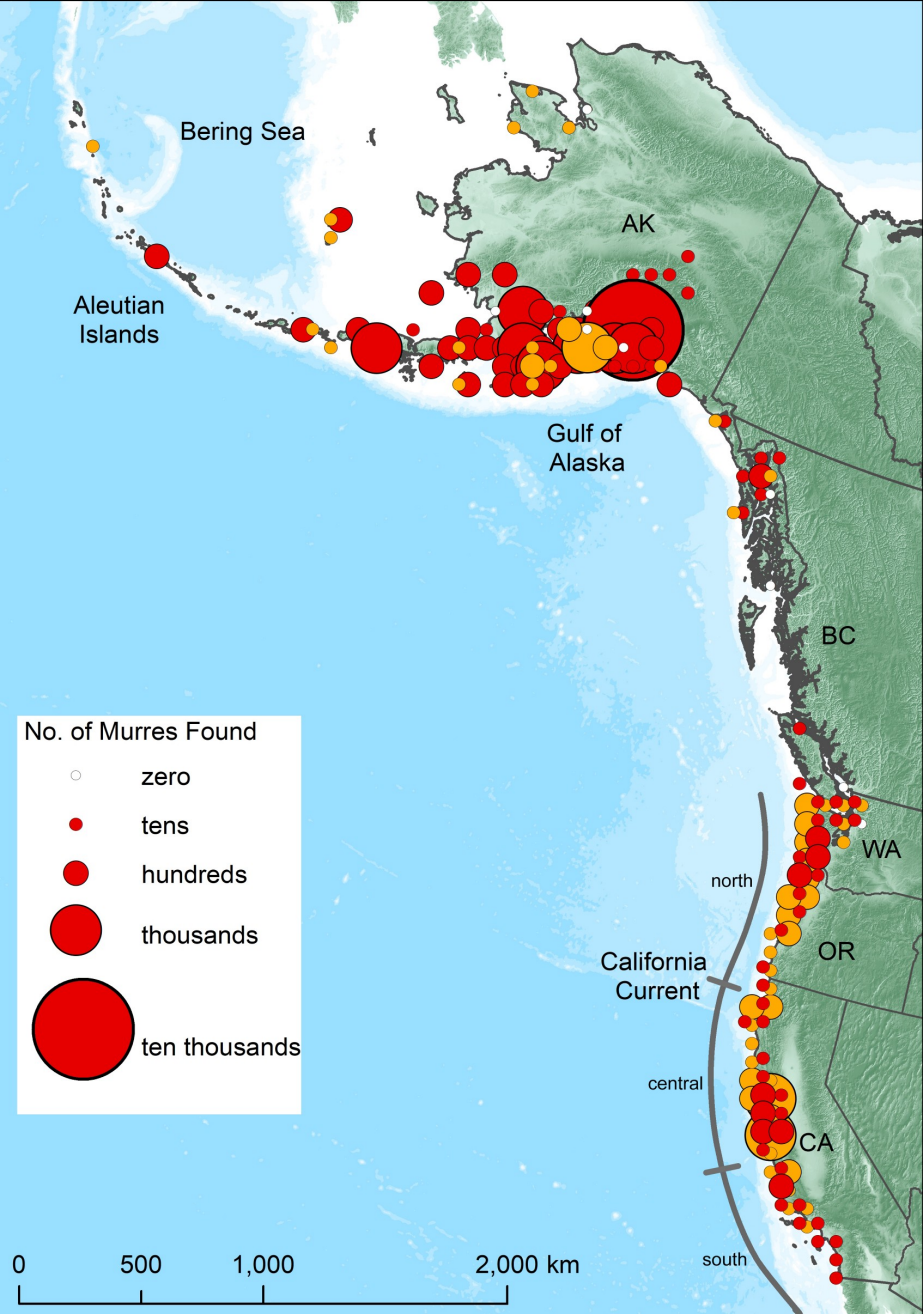
Numbers of dead or moribund common murres observed on beaches that were surveyed systematically (gold circles; ~monthly) and with opportunistic beach surveys and rehab captures (red circles). Areas in which zero dead murres were encountered during surveys are indicated by white circles. All remaining coastlines (without any circles) were not surveyed. Note the California Current System is divided roughly into 3 sections: north (nCCS), central (cCCs) and south (sCCS).

During the event year (May 2015 to April 2016) ~62,000 murre carcasses were reported from a vast range of coastline spanning more than 6,000 km (Table 1, Fig 2). Of these, ~40,000 were obtained from standardized surveys or rehab center reports. Impacts of the heatwave on murres appeared to be most extreme in the northern GOA and the southern CCS. Although few thick-billed murres (*Uria lomvia*) were detected on beach surveys overall (<0.1% of those identified on COASST surveys in Alaska and West Coast), they did comprise a higher proportion of total murres (n = 47) observed on Bering (15%) and Chukchi Sea (86%) beach surveys. Otherwise, the vast majority of murres observed on beaches in the GOA and CCS were common murres.

**Fig 3:**
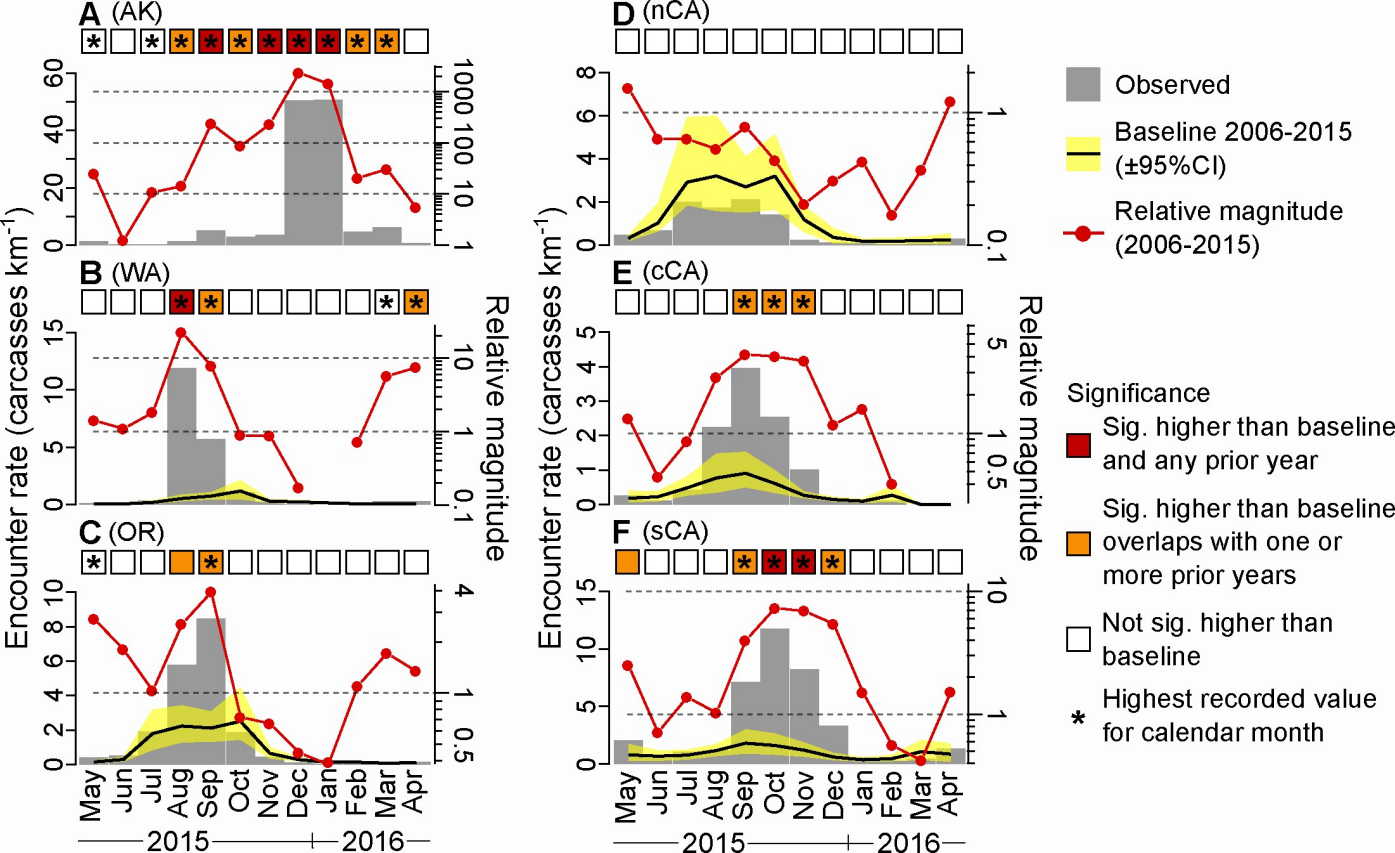
Monthly averaged encounter rates (carcasses per km, gray bars) for the (A) Gulf of Alaska, (B) Washington, (C) Oregon, (D) northern California, (E) central California, and, (F) southern California coastlines. Black lines are baseline encounter rates, yellow shadings are 95% confidence intervals, and red lines show relative magnitude of encounter rates in 2015/2016 compared to the 2006–2015 baseline. Colored squares indicate whether month-averaged encounter rate was significantly higher than baseline and whether they were significantly higher than any prior year of data collection for that calendar month. Asterisks indicate that the mean encounter rate in the corresponding month was the highest value ever recorded. Note that the GOA baseline is so low that it cannot be seen on the chart. Relative magnitude was calculated as the 2015/2016 encounter rates divided by the baseline mean value.

Encounter rates from southcentral California to the GOA were significantly elevated relative to baseline (Fig 3), and this trend was extreme in the GOA, with magnitudes 10x to 1,000x normal for 9 continuous months (Fig 3A). In the GOA month-averaged encounter rates were the highest recorded (relative to monthly baselines: 2006–2014) from May 2015 through to March 2016 (except for June 2015), with the majority representing a statistically significant departure from baseline (Fig 3A). In addition, from September 2015 to January 2016 (except October 2015), month-averaged encounter rates were significantly higher than any previous year of data collection in the Gulf of Alaska (Fig 3A). Numbers counted in other parts of Alaska, including southeast Alaska, and the Bering and Chukchi Seas, were not remarkable (Table 1, encounter rates not shown) but these are vast, scarcely populated areas and COASST sampling was limited. In the GOA, the elevated mortality signal was unusually prolonged, beginning in May 2015 coincident with onset of breeding, and peaking at over 1,000x normal in December 2015 and January 2016, representing an average encounter rate of over 50 carcasses per km. By April 2016, encounter rates had dropped to 10x baseline.

In the CCS, murre carcass encounter rate is typically about 1–3 carcasses per km during late summer and early fall (July-October; Fig 3B–3F) largely due to juvenile mortality following the breeding season. This is markedly higher than the baseline in the GOA, which typically peaks at ~0.1 carcasses per km (Fig 3A). In Washington and Oregon, encounter rates were statistically higher than baseline from August to September of 2015. This represented the highest encounter rates ever recorded for those calendar months in Washington (Fig 3B), although encounter rates weren’t significantly higher than all previous years (S1 Text). In northern California encounter rates were at or below average, and significantly lower in November of 2015 (Fig 3D). In North-Central California, encounter rates were significantly higher than baseline and were the highest on record for September to November (Fig 3E), and into December in South-central California (Fig 3F). However, confidence intervals for these months overlapped with one or more prior years of data collection (S1 Text). Overall, mortality rates were most elevated above average in the north and south extents of the heatwave.

### Necropsies

**Fig 4:**
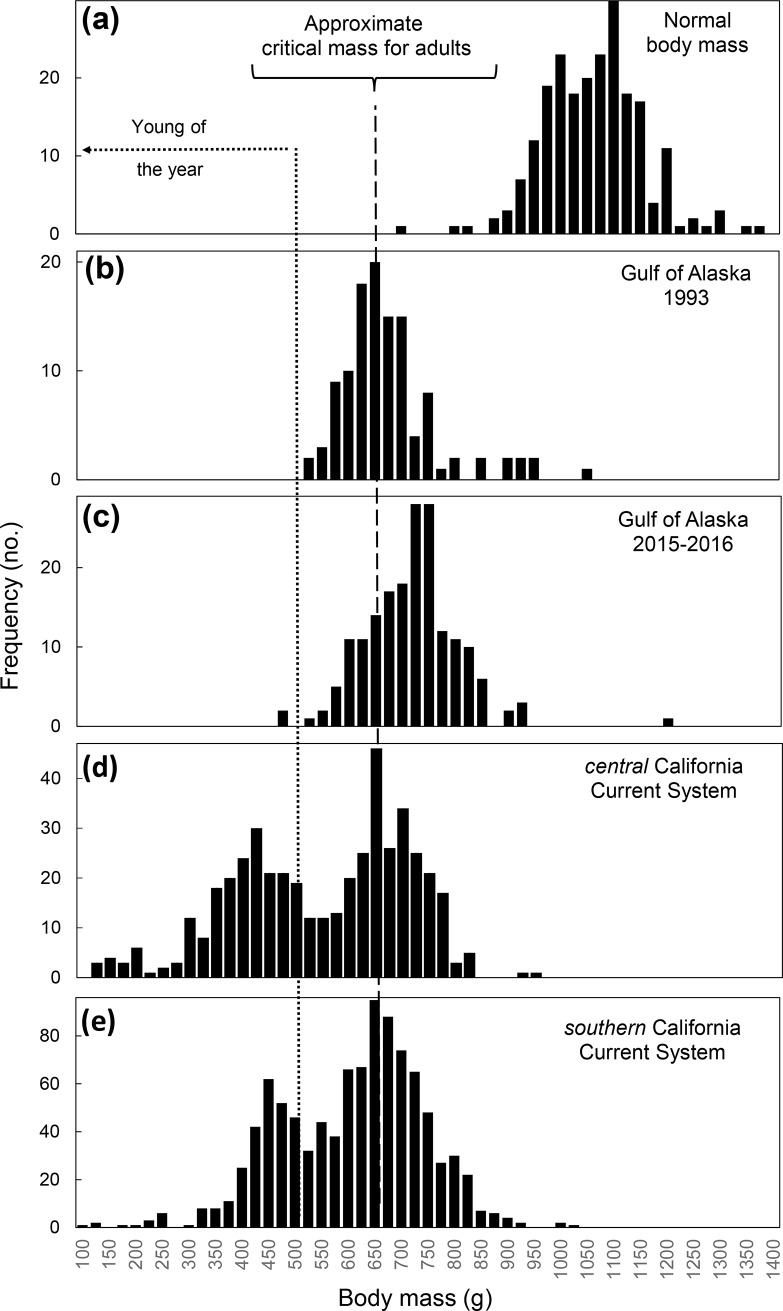
Body mass of common murres collected: (a) at seven breeding colonies in the Gulf of Alaska (GOA), May-September 1988–1999; (b) after a large die-off of murres on the Kenai Peninsula, GOA, February-March 1993; (c) at scattered locations in the GOA and Bering Sea, May 2015-April 2016; (d) along the central coast of California (37°-42° N), May 2015-April 2016; and, (e) along the southern coast of California (32°-37° N), March 2015-April 2016. The vertical dashed line represents the approximate critical mass below which mortality is expected in starved common murres (phase III starvation). The vertical dotted line indicates the cutoff mass below which birds were likely young-of-the-year fledglings.

Of the common murre carcasses collected in Alaska and necropsied at the National Wildlife Health Center, 79% were AHY (68% adult, S2 Table) and 68% were female. All AHY birds were emaciated (mean mass = 711.1g ± 95.0 SD) and severely underweight compared to live healthy birds collected at colonies during the breeding season (n = 219 AHY, mean mass = 1054.0 ±94.3 SD; Fig 4). Emaciation was characterized by moderate-to-severe pectoral muscle atrophy and absence of subcutaneous, epicardial and visceral fat reserves. Emaciation was the most significant postmortem finding contributing to death in the majority of birds necropsied. A few (n = 4) individuals had mild-to-moderate nematode and/or cestode intestinal parasite infections (insignificant to death) and one had septicemic salmonellosis. There was no other evidence of infectious disease. Trace levels of saxitoxin (1.4–3.9 ppb) were detected in 20% of samples (n = 8) tested. Domoic acid was not detected.

From subsamples necropsied at the ASC, we determined that 67% of birds were female, 89% were AHY birds (64% adult), and 11% were HY. Nearly all (97%) birds examined for pectoral condition were scored as emaciated and of those scored for subcutaneous fat most had zero (83%) or very little (17%). Only 7% of birds (n = 100) had food remains (trace amounts, mostly bone fragments) in the gizzard. Mass of all birds (n = 97) averaged 715.2 ±79.9 SD. Overall, body mass of murre carcasses collected during the event year were comparable to that of murre carcasses measured (n = 116 AHY, mean mass = 666.0 ±92.4 SD) during a similar die-off in Prince William Sound in March 1993 [20].

### Rehabilitation birds

Of 3,365 murres examined at rehabilitation centers, 8% were dead on arrival or euthanized immediately; 47% were described primarily as emaciated; 5% were injured in some way (e.g. broken wing); and 5% were oiled. The remainder (35%) had non-specific information (e.g., beached, sick, weak). The frequency of these conditions varied from south to north, with 91–99% of birds classified as “starving” in Oregon, Washington and Alaska, while “other” causes increased in frequency in southcentral California (13%) and northcentral California (25%). Birds received at rehab centers in California exhibited a bimodal pattern of mass distribution (Fig 4), reflecting a large proportion of HY (34% south and southcentral; 55% northcentral and north) birds. Average mass (±SD) of AHY birds was similar to those observed elsewhere (south and southcentral: 682.7 g ±78.0, n = 402; northcentral and north: 675.6 g ±72.0, n = 157; Fig 4).

### Murre reproductive failures

**Fig 5:**
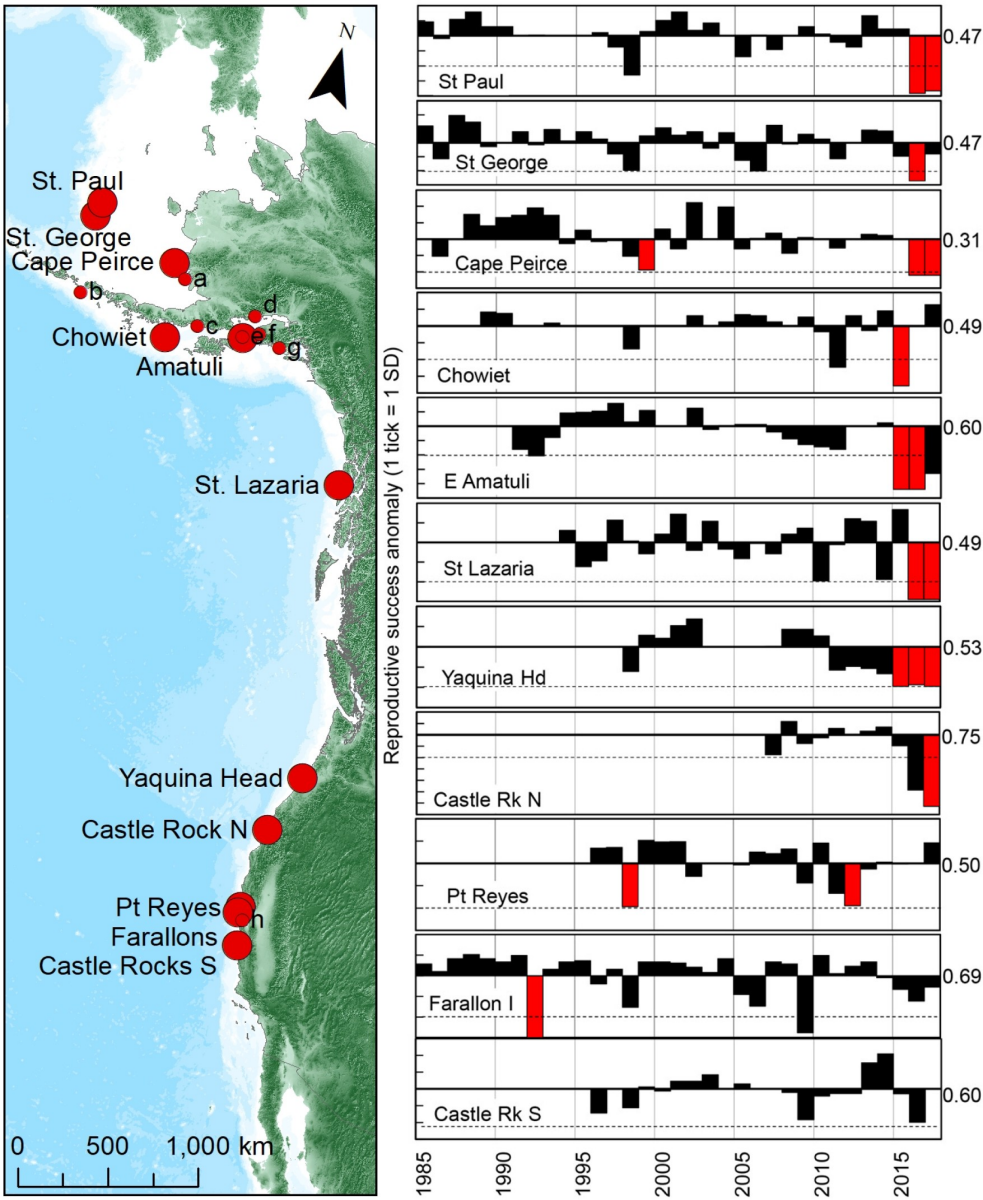
Annual standardized deviations in reproductive success of common murres (on right) at continuously monitored colonies (on left, large red circles) distributed over ~6000 km in the NE Pacific Ocean. Red bars (on right) indicate every known year of reproductive failure (i.e., success 0.0 to <0.10 chicks/pair) from 1985 to 2014. Horizontal dashed lines indicate where reproductive success would fall two standard deviations below the long-term average (for pre-event average during 1996–2014, value at right of each plot). Smaller and/or irregularly monitored colonies (on left, small red circles) included (a) Round I., (b) Aiktak I., (c) Oil Creek, (d) Duck I., (e) Nord I., (f) Gull I., (g) Barwell Is., (h) Devil’s Slide Rock.

Just under one quarter of the Alaskan common murre population resides on colonies regularly monitored for attendance and reproductive success (*rs*) (Fig 5; [40]). Out of 138 colony-years (i.e. sum of colonies monitored times years of effort) at large relatively stable colonies from 1995 through 2014, only one complete (*rs* = 0 chicks/pair [ch/p]) and seven low (*rs* ≤0.10 ch/p), see Methods) reproduction failures have been observed (at Round Island, Cape Peirce). Aiktak, a small (~1200 birds) colony in the eastern Aleutians, is the only monitored colony with frequent reproductive failure (12/21 years), which may be why it has decreased rapidly in size in recent decades [40].

During the 2015 breeding season, two annually monitored colonies (Chowiet, Amatuli) failed completely, as did one occasionally monitored site: Gull Island (pre-2015 mean = 0.54 ch/p). In the GOA, these three sites comprise ~26% of the common murre population. Only two regularly monitored colonies reported an above average *rs* (Fig 5).

In 2016, reproductive failures expanded in the GOA and Bering Sea. Seven of eight annually monitored colonies in the GOA and Bering Sea, and five intermittently monitored colonies in the GOA (Barwell, Nord, Gull, and Duck islands; Oil Creek) failed completely (0 ch/p). Of regularly monitored colonies, only Chowiet in the Semidi Islands (GOA) produced fledglings (0.48 ch/p) but far fewer birds attempted to breed (39% of pre-2015 high count of 4283 murres on monitoring plots). Additional signs of reproductive difficulties during 2016 in the GOA included late egg-laying (if it occurred at all), irregular attendance and total abandonment at some colonies.

Reproductive difficulties continued in 2017, as four out of seven regularly monitored colonies failed completely (Cape Pierce, Round, Aiktak, St. Lazaria), and two others (St. Paul 0.02 ch/p, Amatuli 0.15 ch/p) experienced failure or unusually low success (below 2 SD of long-term mean (Fig 5). In addition, two irregularly monitored colonies (Gull, Duck) failed completely. Only Chowiet performed above its long-term mean (2017: 0.66 ch/p), but again, only 43% of pre-2015 numbers were seen on nesting ledges so total production would be only 28% of pre-2015 numbers.

In summary, 13 common murre colonies in the GOA and Bering Sea experienced a complete failure (0 ch/p) in reproduction at least once during the event years (2015–2017). Multi-year failures were documented in 8 colonies. Out of 31 colony-years of *rs* observed during the event years, 25 (81%) were below their long-term average, 19 (61%) were complete failures (0 ch/p), and 6 more (mean = 0.16 ch/p) were well-below average.

There is evidence that common murres in the CCS also experienced depressed reproductive success, albeit to a lesser degree and somewhat lagged in comparison to Alaska. Reproductive success in the CCS population is usually stable, as informed from three decades of monitoring (1985–2014) at 5 colonies representing ~64% of the CCS population (Fig 5). Reproductive success only fell below 1 SD of the mean in 14% (12/83) of colony-years, and below 2SD of the mean in 5% (4/83) of colony years (Fig 5). In contrast, *rs* fell 1 SD below the mean 50% of the time (7/14 colony-years) during 2015–2017. At Yaquina Head (OR), a colony already depressed by disturbance from bald eagles (*Haliaeetus leucocephalus*) and brown pelicans (*Pelecanus occidentalis*)[41], near (<0.02 ch/p in 2016) or complete failures (0 ch/p) were recorded in all three years. At Castle Rock North, the largest murre colony in northern CA, success plummeted from baseline average of 0.75 ch/p in 2007–2014 to 0.17 ch/p in 2016 (>2 SD below mean), followed by complete failure (0 ch/p) in 2017. At the Farallon Islands, the largest colony in central CA, *rs* was moderate (0.45–0.58 ch/p) in 2015–2017, and consistently below the baseline average (0.69 ch/p), while *rs* at nearby Pt. Reyes was at or above (0.49–0.73 ch/p) baseline average (0.50 ch/p). Finally, at two other small colonies south of the Farallons, *rs* in 2015–2017 was slightly above average (mean = 0.65 ch/p, n = 22 years) at Devil’s Slide—a small colony that was re-established with social attraction—and well-below (~2 SD) the long-term average at Castle Rocks South in 2016 (Fig 5).

## Discussion

### Die-off magnitude and timing

The 2015–2016 common murre die-off in the northeast Pacific is unprecedented globally in magnitude, spatial extent and duration. It occurred during a heatwave that was also severe (Category III) in magnitude, spatial extent and duration (711 days, [10]). The relative impact was greatest in Alaska, where ~47,000 carcasses reflected encounter rates that were up to a thousand times higher than usual. Peak encounter rates topped 4,600 carcasses/km in Prince William Sound. Many (~14,500) birds were also found on West Coast beaches, but part of this total resulted from much larger beach survey and rehabilitation efforts (S1 and S2 Figs). About one-third of all birds counted on West Coast beach surveys (~11,800) can be accounted for by average background mortality in the region, and about three-quarters of the above-average (~5-10X) mortality was concentrated in the southern California Current System (CCS) (Fig 3). Although strong heatwave anomalies occurred throughout the ~6000 km spatial range over which murres died, highest mortality rates occurred along an ~1,000 km arc of coastline in the northern Gulf of Alaska (GOA) and an ~500 km stretch of coastline in the southcentral and southern CCS, areas that overlapped spatially with the strongest SST anomalies and most rapid rates of warming (Fig 1).

To put numbers into perspective with other mass mortalities in Alaska, biologists counted 22,800 emaciated (average 704 g) murre carcasses along an ~700 km stretch of coast on the southeast Bering Sea following a severe storm during April 1970 [42]. Aerial surveys averaged 80 carcasses/km (maximum 5440 carcasses/km) and total mortality was estimated conservatively to exceed 100,000 birds. Following the *Exxon Valdez* oil spill in March 1989, ~30,000 seabirds (74% murres) were recovered along a ~750 km stretch of coast in the northern GOA [43]. Based on a variety of *in situ* experiments to determine how many carcasses made it on shore and were likely to be counted (see below), models predicted that 300,000 to 645,000 birds actually died at sea [44,45]. In March 1993, about 3500 dead murres were recorded on beaches in the northern GOA; all were severely emaciated (Fig 4). Deposition and persistence rates of murres on beaches were calculated from repetitive surveys [26] which indicated that a total of 10,900 murres were deposited cumulatively on the beaches surveyed. Assuming very conservatively that 90% of birds at sea came ashore, and that 10% of beaches in the die-off region had been surveyed, it was estimated that ~120,000 murres died in this wreck [20].

Few birds were recovered on beaches in British Columbia or southern southeast Alaska (Fig 2), but this is a notable gap area in the distribution of murres during both summer and winter [46] (S3 Fig). In addition to a scarcity of murres, this area is sparsely populated and there was little search effort there (Fig 2). Along the U.S. West Coast, murres are widely abundant (S3 Fig) and one of the more common species recorded on beach surveys, especially juvenile murres after they depart colonies in late summer [22,25,47]. However, there are few historical reports of natural die-offs involving more than hundreds or low thousands of birds, or of adults in particular. The recovery of ~8100 carcasses above the baseline is unprecedented for a “natural” die-off on the West Coast but has been surpassed in magnitude by the mortality of tens of thousands of murres in oil spills [46].

A few exceptionally large die-offs have also occurred elsewhere in the world. During winter of 2013–2014, a total of 54,982 seabirds, mostly (54%) Atlantic puffins (*Fratercula arctica*) and common murres (29%), came ashore from Portugal north to the Shetland Islands, but mostly (80%) along the French coast [48]. This number “is likely to be a large underestimate of the final death toll.” Most mortality was attributed to starvation, perhaps precipitated by a powerful storm and difficulties foraging. There was no heatwave happening at the same time, but the die-off followed a nearly 30-year increase in SST in the North Atlantic from the 1980s through 2000s. This long-term increase in ocean temperature was implicated in the decline of several seabird populations during this period, as well as a reduction in abundance and quality of some forage fish species [39,49–52]. Elsewhere, following a major heatwave in the Tasman Sea [53] and after a severe winter storm off New Zealand in 2011, more than 53,840 dead prions (80% broad-billed prions *Pachypatila vittata*) were counted on long-term survey beaches during July and August [54,55]. Carcasses were found over the entire west coast of New Zealand and densities exceeded 1000 birds/km on several beaches. Total mortality was estimated conservatively at 250,000–500,000 individuals.

Counts of dead seabirds on beaches following mass mortality incidents represent a minimum measure of total mortality. They do not include carcasses that sink at sea, or those washed ashore that are removed by scavengers or buried in sand and debris. Furthermore, the frequency and thoroughness of beach surveys ultimately determines how many carcasses will be discovered and counted [14,44,56]. Experimental studies (n = 19) conducted by releasing marked alcids (and/or decoys) at sea when systematic beach surveys were underway indicate that under a wide range of conditions at least 6.9x (95% CI 4.3x to 14.2x) more birds die at sea than are found on nearby beaches [57]. Recovery rates ranged between 0% and 61%, and much depends on the specifics of every experiment (e.g. wind direction, extent of search effort, etc.). Actual mass mortality events exhibit a range of expansion factors of similar magnitude (e.g., Tasman Sea 5x-10x [54]; Gulf of Biscay 5x-17x [57]; Gulf of Alaska in 1989 10x-22x [44,45]) or larger magnitudes (e.g., Gulf of Alaska in 1993 34x [20]; Gulf of Mexico 80x-950x [58]; Bering Sea 579x [59]). The largest multipliers were attached to studies of prolonged mortality (e.g. DeepWater Horizon oil spill, [58]) or those estimated by extrapolating from transects at sea (e.g. [59]).

In this study, owing to logistic constraints and geographic expanse, we measured few or none of the factors needed to model total mortality in the areas most affected. However, we can draw upon a comparable study of carcass counts [45] conducted after the 1989 *Exxon Valdez* oil spill (EVOS) in which rates of carcass sinking, deposition, persistence, and search effort, were all measured in the core area of the oil spill zone [43], which also happens to overlap considerably with the area of highest murre mortality during the 2015–2016 heatwave. If we apply expansion factors determined from that study to bracket the lower (10x) and upper (22x) estimated limits of total mortality in the heatwave, we estimate that between 470,000 to 1,030,000 birds died in the Gulf of Alaska during the heatwave. This total probably included birds overwintering from Bering Sea colonies (see below), and it suggests that as much as one quarter of all murres breeding in the Gulf of Alaska and southeast Bering Sea (~4.5 million, U.S. Fish and Wildlife Service colony estimates and correction factor for birds at sea [46]) might have been killed.

On the West Coast, we don’t have a comparable model to estimate total mortality. However, we know that beach survey coverage was more comprehensive on the West Coast, and so we used the conservative range of expansion factors from experimental studies (above, [57]) to estimate that 4x to 14x more birds than the ~14,500 murres counted were killed, i.e., between 58,000 and 203,000 birds. This would comprise about 4 to 14% of the CCS population (~1.5 million, estimated as above).

Taken together, the total impact of the heatwave on common murre populations throughout all areas was likely between 0.53 and 1.2 million birds, or approximately 10–20% of total populations (~6 million). The fact that most birds killed in the die-off were probably breeding adults compounds the seriousness of the mortality for the population [60], and it will take longer for recovery of the population than if the die-off had affected mostly juveniles [60,61].

### Reproductive failure at colonies

The extreme reproductive failures of common murres that occurred during summer 2015 and in the two years after the main die-off were also focused in Alaska and occurred less frequently in the California Current System (CCS). Considering the low number of birds encountered on beaches in the Bering Sea, breeding failures at colonies there were surprisingly similar in magnitude to those in the Gulf of Alaska (GOA). This reduction in juvenile production will significantly delay recovery of populations in all affected colonies [60]. Also, the number of birds attending colonies in the Bering Sea (data from USFWS [40] and USGS averaged in 3-year windows before and after the 2015–2016 die-off, weighted by colony size) declined more in the Bering Sea (>80%) than in the GOA (>50%). Whether these declines were due to reduced attendance because of deferred breeding [62], or a crash in colony populations due to a crash in food supply [61] is still not clear. Either way, reproductive failures and reduced attendance in the Bering Sea suggest that prey deficits were also experienced by murres in the southeastern corner of the Bering Sea.

The frequency of total reproductive failures (n = 22), overall reduced breeding success and decline in numbers that occurred at multiple colonies in the northeast Pacific during 2015–2017 is a cause for astonishment and alarm. The common murre is probably the most widely studied seabird in the Northern Hemisphere and total reproductive failures at well-established colonies have been rare during some 70+ years of detailed observations (Fig 5)[17,39,40,46,63]. A smaller-scaled but similar die-off of murres in association with a collapse of forage stocks (capelin, sand lance, juvenile Atlantic cod *Gadus morhua*) occurred in the Barents Sea in 1986 [61]. Large common murre populations at many colonies in that region subsequently declined by 60–95% in a single winter [64]. Recovery of forage stocks and murre population growth started in the next year. However, two decades passed before murre populations recovered to pre-crash levels [61]. It remains to be seen when (or whether) murre populations in Alaska will recover from the heatwave in light of predicted global warming trends and the associated likelihood of more frequent heatwaves [5].

### Causal factors

Several acute biological responses to this unprecedented heatwave were observed throughout the northeast Pacific. Phytoplankton biomass in the northeast Pacific transition zone waters was lower in winter 2014 than in any year measured since 1997 [11]. The largest and most wide-spread harmful algal bloom in recorded history—a bloom of *Pseudonitzschia*—extended from California to the Gulf of Alaska (GOA) in 2015 [12,13]. Fundamental shifts in coastal productivity indices [11] and micronekton assemblages [65] were also associated with this sustained warming event. Large predatory groundfish in Alaska, including trophically and commercially dominant species such as walleye pollock (*Gadus chalcogrammus*), Pacific cod (*Gadus macrocephalus*), arrowtooth flounder (*Atheresthes stomias*) and Pacific halibut (*Hippoglossus stenolepis*) all declined in body condition and some in abundance (e.g., cod, see below) during heatwave and post-heatwave years of 2015–2017 in the GOA and Bering Sea [66–69]. A large die-off of planktivorous Cassin’s auklets (*Ptychoramphus aleuticus*) occurred from central California to British Columbia (BC) in the winter of 2014–2015 [14] followed by a large die-off of rhinoceros auklets (*Cerorhinca monocerata)* in the same region during 2016 [70]. Hundreds-to-thousands of young-of-the-year California sea lions (*Zalophus californianus*) died in 2014 and 2015, and Guadalupe fur seals (*Arctocephalus townsendi*) died in large numbers and experienced reproductive failures during 2015 [15,71,72]. A record total of 79 humpback and fin whales stranded during 2015–2016 in Alaska and British Columbia waters, mostly for “unexplained” reasons, and mostly in the GOA [16]. This was accompanied by a >50% decline in summer populations of humpback whales, evidence of malnutrition (“skinny whales”), and near complete absence of calves in Glacier Bay between 2014–2017 [73].

A common thread to most of these events was that they involved either a loss in productivity or a mass mortality of higher trophic-level animals, both of which point to problems in food production or availability. All the vertebrate predators affected also share a common dietary dependence on a few key forage species (see below) and this points to a bottleneck in the forage base. These events all occurred within, and for some years after, the time-frame of the 2014–2016 heatwave, and over an enormous spatial range involving three large marine ecosystems (CCS, GOA and Bering Sea). This calls for an explanation that is plausible for all species and regions, and that involves water temperature as a driving force—either directly or indirectly. With respect to murres, we offer three non-exclusive hypotheses to explain the cause of these events: 1) temperature-mediated changes in the distribution and quality of the prey base available to murres; 2) harmful algal blooms associated with warm water anomalies; and, 3) temperature-enhanced competition from ectothermic predators.

#### Bottom-up effects: Murres as marine predators

Reduction in primary production, and ultimately zooplankton or forage fish biomass, has been implicated in past seabird die-offs and reproductive failures (e.g., [14,27,46,61,74–78], often in association with anomalous oceanographic conditions (too warm, too cold, loss of upwelling, etc.). In order to understand how murres are affected by climate-mediated bottom-up changes in their forage base, we need to first consider their foraging ecology and the types of prey they eat. Throughout their Pacific range, common murres feed on a wide variety of prey, but around any particular colony they select among just a few species that may be found nearby such as sand lance (*Ammodytes personatus*), capelin (*Mallotus catervarius*) and other smelt, Pacific herring (*Clupea pallasii*), Pacific sardine (*Sardinops sagax*), northern anchovy (*Engraulis mordax*) and euphausiids (e.g., large *Thysanoessa* species), as well as juvenile age classes of salmon, gadids, hexagrammids, rockfish and squid [79–83]. (Note, we lump euphausiids and squid with “forage fish” here because a few invertebrates are also consumed in abundance by “piscivorous” groundfish, seabirds and marine mammals, especially in winter). Common murres are extremely well adapted for foraging on continental shelves; they fly faster than any other northern seabird [84], are capable of traversing any shelf in the CCS or GOA within hours, and, they are deep divers, making the entire shelf habitat accessible [85]. This is probably why breeding failures and die-offs have been historically rare (see above).

On the other hand, as endotherms living in hostile, cold environments, murres maintain high metabolic rates (2.14 kJ/g/d [86]) and assuming an average body mass of 1054 g in the GOA (Fig 4) and a base energy value for “high quality” prey (5.0 kJ/g wet, [87]), murres need to eat 56% of their body mass every day to meet daily metabolic demands. Murres in Alaska generally eat age-0 or age-1 forage fish that weigh approximately 5–10 g ([88–90], J.F. Piatt and M. Arimitsu, unpubl. data), so to maintain body mass, murres would have to catch and eat about 60–120 high-lipid forage fish every day. If only smaller or leaner prey (e.g., juvenile pollock) were available, then the number needed could double [87,91]. By comparison, an ectothermic cod of similar size to a murre would only need to eat about 0.4–1.5% of its own body mass (BM) in food per day [92], i.e., as little as 1–3 high-quality forage fish a day.

This is the ultimate “Achilles heel” for murres, and one that sets it far apart from competing ectothermic groundfish (eating typically 0.1–1% of BM/d) and endothermic marine mammals [93] including large cetaceans (1–2% BM/d) or small cetaceans and pinnipeds (5–15% BM/d). If murres can’t fully meet this food demand every day, they lose body condition quickly and jeopardize survival. If they can’t find *any* food for 3–5 days, they will die of starvation [36]. The fact that common murres are the most successful and abundant piscivorous seabirds breeding in the Northern Hemisphere speaks to their remarkable ability to meet this demand day-after-day. However, shifts in taxonomic composition of prey fields in response to changing environmental conditions have been shown to dramatically reduce murre foraging success, reproductive success and survival occasionally [61,83,94], demonstrating that even these superlative marine predators have limits [38,95]. Still, examples of such limitations in murres are rare and the magnitude of the events reported on here are beyond extraordinary.

#### Bottom-up effects: Shifts in the prey base

Warming of subarctic shelf waters may lead to both vertical (deepening) [96,97] and northward migrations of these forage species, or entire communities, according to thermal gradients and tolerances [98–100], a phenomena widely observed during the 2014–2016 heatwave [14,101–103]. In the California Current System (CCS), shifts in zooplankton [101] and forage fish diversity [65,103] signaled a persistent northward expansion of southern species. Zooplankton shifts may have also resulted in a depleted food chain in terms of relative energy transfer [101], although larval forage fish species were actually more abundant in samples off central California and central Oregon [65,103]. In contrast, anchovies and sardines in the CCS both declined by 2–3 orders of magnitude from the mid-2000s to 2014 [104,105]. Although these declines preceded the heatwave, fish and plankton net sampling in Oregon and Washington indicate that catches of these forage fish, as well as of euphausiids were further depressed in 2015 and 2016 [9,12,70,71].

In the Gulf of Alaska (GOA), a similar introgression of smaller zooplankton was observed, along with a breakdown in established SST-phytoplankton-zooplankton dynamics after 2013 [102]. Shifts to earlier peak biomass of smaller copepods associated with warmer temperatures [102] were cited as potential factors in concomitant declines in forage fish quality in Prince William Sound in the winter of 2015–2016 [106,107]. Shifts in forage fish availability in the GOA were apparent in marine bird diet starting in 2014 [108], with a sharp decline in capelin and an increase in sablefish (*Anoplopoma fimbria*), combined with a slow rebound of sand lance. In GOA waters, sand lance began a long steady decline in the early 2000s, to a low in 2011, and remained low to 2015 [67,109]. Capelin stocks were depressed after the 1976 regime shift [110], rebounded dramatically in 2007 as the GOA entered a new cold phase [67,109], and collapsed again during the heatwave in 2014–2016 [67,100]. In 2015 and 2016, age-0 pollock larvae in the GOA were 2–3 orders of magnitude less abundant than average; indicating complete recruitment failures for pollock [67,68]. In sum, latitudinal shifts in zooplankton and forage fish prey, combined with overall depression of major prey taxa, apparently created marginal foraging conditions for murres for several years.

**Fig 6:**
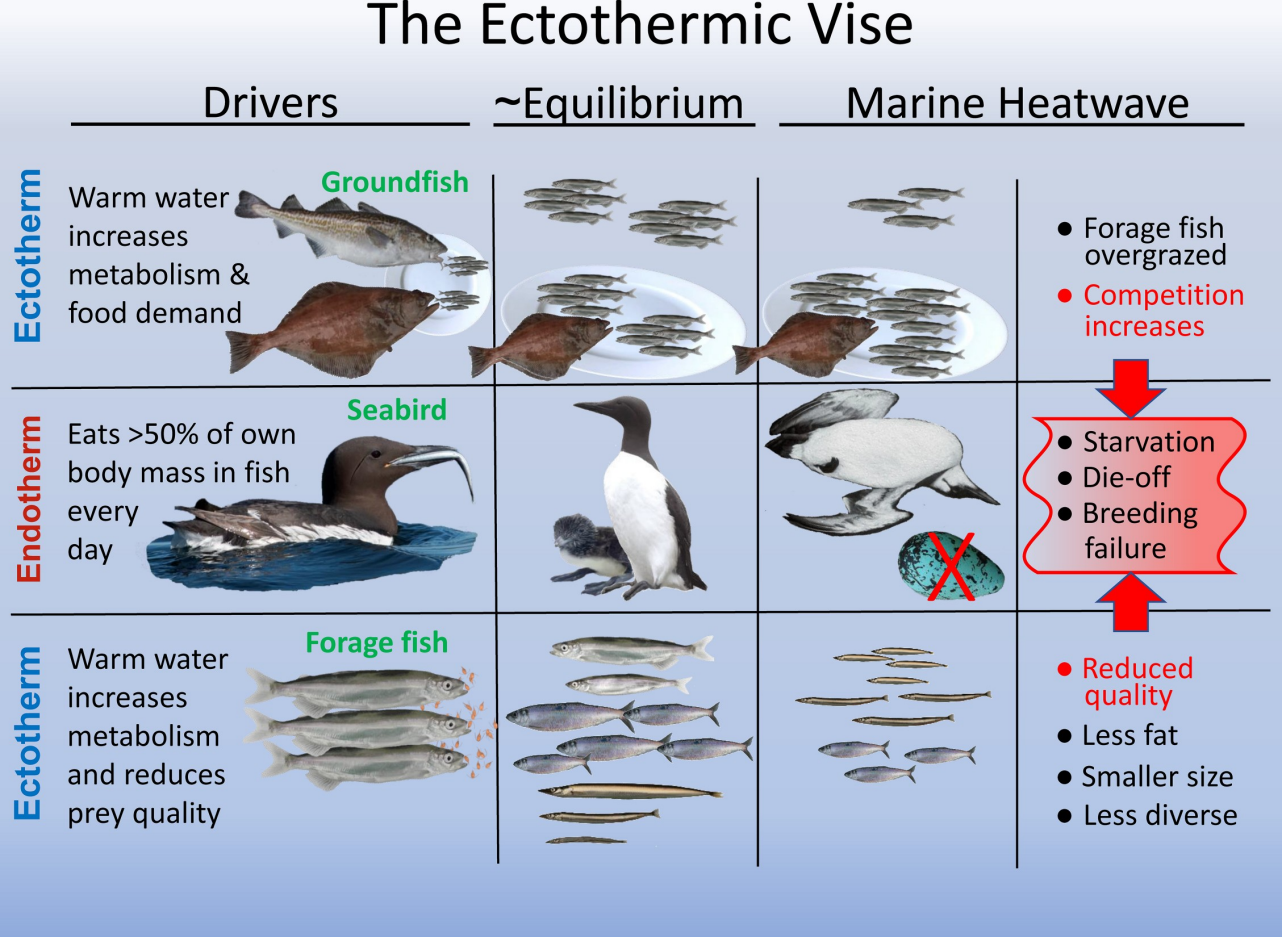
Illustration of the “ectothermic vise” hypothesis to explain the dramatic decline of forage fish and starvation of murres across three large marine ecosystems during the 2014–2016 marine heatwave. We propose that an unusually warm layer of water in the NE Pacific, persisting for more than 2 years, had a powerful cumulative effect on ectothermic groundfish (stimulating food intake rate) and ectothermic forage fish (reducing their quality) leading to a strong top-down and bottom up (vise-like) impact on murre survival and reproductive success.

In addition to shifts in latitudinal abundance of specific taxa, warm water conditions diminished body condition and somatic growth of ectothermic forage fish. Body condition of capelin and sand lance in the GOA and CCS was reduced during the heatwave, resulting in smaller, less energy-dense prey for murres [107,111–113]. Whole-body energy content of age-1 sand lance declined by 44% in 2015 and 89% in 2016 in Alaska [114], and body condition of sand lance in the northern CCS declined markedly in 2014–2015 [113]. Presumably, consumed food was re-directed to fuel metabolism rather than somatic growth or fat storage [69,115]. Similarly, others [116] have shown marked reduction in growth of several CCS forage species during warming events, including the 2015–2016 heatwave. These included northern anchovy, Pacific herring, Pacific sardine, surf smelt (*Hypomesus pretiosus*), and whitebait smelt (*Allosmerus elongatus*), all common prey for murres in the CCS. They also demonstrated a marked change in forage fish diets in 2015–2016, from energy-rich plankton species to energy-poor gelatinous species; a change they ascribe to a restructuring of nektonic communities that occurred in response to the heatwave [116]. Thus, the heatwave increased metabolic demand of forage fish while at the same time it reduced the quality of some prey eaten by forage fish, creating a bottleneck for mass/energy flow to higher trophic levels, including seabirds (Fig 6). As all fish and invertebrates are ectothermic, this effect could potentially have far-reaching impacts on food webs in the GOA and CCS [69,117].

#### Bottom-up effects: Toxigenic algae

Increased ocean temperatures during and following the heatwave have been associated with harmful algal blooms (HABs) [13,118], which are known to cause marine bird mortality, primarily through plankton-derived toxicants entering the food chain and occasionally resulting in die-offs of thousands of birds [119]. Saxitoxin and domoic acid have been widely detected in top marine predators [31,120] but we know little about toxicity levels or effects of chronic exposure in most cases.

During the common murre mass mortality event, an extensive HAB of a toxigenic diatom (*Pseudo nitzschia* sp.) that commonly produces domoic acid was documented in coastal California from March through June 2015 [13] resulting in bioaccumulation of domoic acid in northern anchovies (*Engraulis mordax*), one of the main prey species of common murres [121]. Investigators [120] detected low levels of domoic acid in tissues of beach cast common murres during and after the 2015 bloom (July—November). Nonetheless, they concluded that starvation “was likely the ultimate cause of death” and that any harmful algal bloom effects were secondary. In Alaska, it remains unclear whether HABs played any role in the elevated mortality rates of common murres during the 2015–2016 heatwave. Immediate testing for domoic acid in murres was minimal (n = 9 birds) and none was detected, but *Pseudo nitzschia* were 2-3X more abundant than average on the GOA shelf during 2014 and 2015 (S. Batten, pers. comm.). Saxitoxin, which can cause paralytic shellfish poisoning, has been linked to mortality of seabirds in Alaska [122] and concentrations of saxitoxin in some areas peaked during the summers of 2014–2016 [118]. Trace levels of saxitoxin (1.4–3.9 ppb) were detected in eight of 39 murre samples (stomach or cloacal content) obtained by the National Wildlife Health Center and tested immediately in 2015–2016 (see Methods). Later analyses of an additional 56 murres at the USGS Alaska Science Center, including die-off and healthy specimens, as well as samples of forage fish and invertebrate prey collected in 2015–2017, revealed a low to moderate frequency (20%-54%) of saxitoxin occurrence among taxa groups; but all at low concentrations [123]. Domoic acid was found in a single bird, and in some prey taxa (4%-33%). Authors noted that all biotoxin values were below levels reported in other seabird die-offs where causal links were established between toxin concentration and bird mortality, and as such, do not support a hypothesis that algal bloom biotoxins were a primary cause of murre mortality in Alaska [123].

Furthermore, the likelihood that HAB toxins were a primary and acute causal factor in the die-off appears small given that the center of the murre mass mortality event was the GOA, and the extended duration of the die-off (9 months of 100x baseline) both preceded and extended well past peak HAB bloom windows [118]. Also, we should have seen behavioral changes in affected birds as well as a larger number of species affected if HABs were a primary source of mortality [120,124]. Nonetheless, we are still lacking in basic information (e.g., what is a lethal dose?) about HAB effects on marine birds, and it cannot be ruled out as a contributing factor to the die-off [123]. We need more information on the depuration rates of HAB toxins, acute toxic levels (e.g., LD_50_) and the effects of chronic toxin exposure in order to fully assess their potential contribution to the die-off [120,123].

#### Top-down effects: Resource competition from ectotherms

In addition to affecting spatial distribution of large predatory groundfish [125], increasing water temperature has the immediate and predictable effect of increasing metabolic rate, and usually food demand, of these marine ectotherms when they are operating within preferred temperature regimes [92,126]. The influence of this ecological “master factor” [127] on groundfish must have been substantial during the extreme 2014–2016 heatwave, but this pathway of upper trophic impact has been largely overlooked as a factor in regulating populations of groundfish, or other marine predators that compete with groundfish for food [92,117,128,129].

Recent modeling [92] of the effect of temperature on metabolic rate and food consumption in the GOA of three dominant groundfish predators including walleye pollock (*Gadus chalcogrammus*), Pacific cod (*Gadus macrocephalus*) and arrowtooth flounder (*Atheresthes stomias*) showed that an increase of 2°C in the GOA from pre-heatwave (1981–2011) temperatures would have increased food consumption of these species by 70%, 34% and 65% respectively. If we weight each species consumption estimate by its population size (stock biomass [130]), then the increase in prey demand by all species combined would be 63% higher than it was before the temperature increase. Pacific halibut (*Hippoglossus stenolepis*) show a similar response to increasing temperature in the GOA and Bering Sea [69].

The micronekton most commonly eaten by these groundfish include several species also favored by murres and other avian piscivores, especially capelin, sand lance, juvenile pollock, herring and euphausiids (especially in winter) [131]. Given the size of these three groundfish stocks in 2015 (4.48 M mt, [130]) and calculated consumption rates [92], these groundfish would have consumed ~10 M mt/yr of prey in 2015 if temperatures remained average. By comparison, total annual forage consumption by the ~2.5 million common murres in the GOA (calculations following [46,132,133]) total only ~0.45 M mt/yr. Thus in 2015, without a temperature increase, predatory groundfish would have consumed approximately ~20 times more total prey biomass, and ~6 times more forage fish biomass than murres (since fish comprise about a quarter of these groundfish diets; [134]). The 2ºC increase in water temperature would have pushed ectothermic groundfish prey consumption to ~15 M mt/yr, and thereby substantially increase forage fish grazing rates. Given that groundfish typically out-consume seabirds by 10:1 or even 100:1 ratios in northern shelf ecosystems [135,136], a 60% increase in consumption rates by groundfish should have some consequences for seabirds. No comparable modelling of temperature impact on metabolism of fish in the CCS has been undertaken, but the CCS shelf sustains at least 3.1 M mt of large predatory fishes (mostly hake *Merluccius productus*, rockfish, flatfish] [137,138], comparable to biomass density in the GOA and likely to also have provided significant increases in competition with murres during warming events.

If the GOA marine ecosystem was operating at “relative equilibrium” [139,140] prior to the heatwave, then we hypothesize that this massive increase in foraging rate would have eventually led to prey deficits [69,136] for the groundfish themselves (creating intra-specific competition) and for other competitors such as seabirds and marine mammals (creating inter-specific competition) [136,141]. In this scenario, murres would be more sensitive to reductions in key forage fish species than competing groundfish, which typically have much broader diets and less sensitivity to fluctuations in any one prey type [136,142]. Also, it would presumably require a passage of some time for elevated grazing to deplete prey stocks below critical levels needed by murres. In fact, it was almost a full year from start of the “official” heatwave (August 2014, [10]) and more than 3 years after the rate of warming turned positive in 2012 (Fig 1) before a few murre colonies experienced reproductive failure and elevated murre mortality appeared in the GOA and northern CCS (Figs 3 and 5). Murre mortalities increased and persisted through fall in the GOA and southern CCS, and then peaked in the GOA during December 2015 and January 2016, a full 18 months after initiation of the heatwave. Murre die-offs diminished to background levels by April-June of 2016, as water temperatures returned to normal [10]. In contrast, reproductive failures peaked at 13 colonies during the summer of 2016 (23–24 months from heatwave initiation), continued at 9 colonies in 2017, and declined to only 4 colonies in 2018 (USGS, USFWS unpubl. data), although it is bracing to remember that a synchronized failure of even 4 murre colonies would have once been considered an extreme event. Overall, these findings suggest that prey stocks were replenished slowly during the 2 years after the heatwave ended in summer 2016, or that some sort of relative equilibrium among ectothermic and endothermic predators was being re-established following a large cull of bird, fish and mammal populations, or both.

While murres were visibly dying *en masse* and failing to reproduce in 2015–2017, adult Pacific cod populations in the GOA were silently crashing underwater. Following three years in which commercial catches were well below quotas in the GOA (2015–24%, 2016–35%, 2017–45%), and a severe reduction in abundance of some older age cohorts occurred, the allowable catch quota for 2018 was reduced by 80% from the 2017 level [129]. The decline in the cod stock was attributed to reduced adult survival from starvation owing to a major reduction of forage in diets (especially capelin), coupled with a large increase in metabolic rates and food demands [129]. The same changes appeared in arrowtooth flounder, to a lesser degree [69,143]. In addition, the authors concluded that “other ectothermic fish species would be expected to have similarly elevated metabolic demands during the warm conditions, increasing the potential for broad scale prey limitations”, a conclusion that would seem to fit Pacific halibut in the Bering Sea and GOA as well [69]. Indeed, we might expect elevated consumption from some more of the other 30 commercial groundfish species [130], five species of salmon, and non-commercial fish species, alongside the usual competition from other common *endothermic* piscivores in the GOA, including at least thirty other species of seabirds, ten cetaceans, and five pinnipeds [144,145]. Finally, we find independent supporting evidence for potential basin-scale resource competition between large predatory fish and seabirds in the Bering Sea, where biennial high-low cycles in pink salmon (*Oncorhynchus gorbuscha*) abundance result in high rates of forage consumption in high salmon years, and create synchronized biennial cycles in seabird body condition and reproductive success (both low in high salmon years [141,146,147]).

#### Summary- effects of an ectothermic vise

During the most powerful marine heatwave on record [10], an extreme die-off and chronic breeding failure of common murres in the NE Pacific resulted from a widespread shortage of forage fish across three large marine ecosystems. Major impacts on murres included a large reduction (possibly 10–20%) in the breeding population and a severe reduction in productivity that will dampen recruitment for several years. Food shortages were also documented during the 2014–2016 heatwave in many other piscivorous marine predators including groundfish, seabirds and marine mammals. And finally, a variety of ichthyoplankton and pelagic fish studies provided direct evidence for major forage fish declines during 2015–2016 in many of the key species eaten by murres from California to the Bering Sea.

Our hypothesis to explain the wide-spread depletion of forage fish is based on three facts: 1) metabolic rate, food intake rate, somatic growth, fat storage, fecundity and survival in marine ectotherms are strongly modulated by water temperature, 2) these physiological and life history traits are adapted to function optimally over relatively small ranges of water temperature that are species-specific, and, 3) physiological efficiency declines markedly when ambient temperatures wander too far above or below the optimal temperature range. This is why fish seek out water with temperatures that optimize these parameters, e.g., by migrating vertically in and out of warm surface layers or migrating geographically to stay within waters that suit their tolerances and/or facilitate various activities (e.g., migration, foraging, predator avoidance, “hibernation”, etc.). Such effects were noted widely in zooplankton, ichthyoplankton, forage fish and groundfish during the heatwave. However, murres and many other marine predators are highly mobile and deep-diving, and it is unlikely that simple distributional changes in forage fish can account for all the widely observed starvation, mortality and breeding failures in murres.

A more plausible hypothesis is that persistently warm water temperatures modulated ectotherm physiology everywhere at the same time, and forage fish were caught in an “ectothermic vise” (Fig 6). At lower trophic levels, warm waters changed zooplankton community composition, sometimes by the loss of key high-lipid species, sometimes by immigration of less nutritious warm-water species, or both. Consequently, the flow of energy to forage fish was disrupted, even as their own metabolic demand increased. This likely led to reduced somatic growth and fat storage [69,107]. In turn, this reduced survival in some forage fish (or age-classes) and lowered the nutritional quality of forage fish for seabirds (Fig 6). At higher trophic levels, the stimulation of metabolic rates in larger predatory fish led to a huge increase in their intake of forage fish [69,92]. This, in turn, led to a steady depletion of forage stocks for the 2-year duration of the heatwave, and an increase in competition for a dwindling supply of forage (Fig 6).

An ectothermic vise on forage fish ought not to be expected under every scenario of ocean warming, and much may depend on how well community thermal optima are aligned with temperature regimes before, during and after a heatwave occurs [92,117]. Still, it might be useful to model metabolically regulated trophic relationships within marine communities under future warming scenarios in order to assess the potential impact of future warming events on marine food webs. As noted by John Bruno et al. [117] “temperature-driven changes in metabolism play an important and informative role in controlling many of the patterns and processes that interest ecologists. A small but growing body of research suggests that the effect of temperature on marine populations and communities is at least as strong as other factors that receive far greater attention, e.g., competition, predation, and resource availability.”

## Supporting information

S1 Table Wildlife rehabilitation organizations contacted in California, Oregon, Washington, British Columbia and Alaska. (DOCX)

S2 Table Common murre collection details and measures of mass, sex, and age class. (DOCX)

S1 Fig Distribution and numbers of live birds received at 37 rehabilitation centers. (TIF)

S2 Fig Locations of individual surveys conducted for beached birds during the period May 1, 2015 to April 30, 2016. (TIF)

S3 Fig Summer and winter distribution of Common Murres in the NE Pacific. (TIF)

S1 Text Methods for bootstrap calculation of significance in testing rates of carcass encounter on beach surveys in different months and years. (DOCX)

S1 Dataset Data collected on common murres taken in by bird rehabilitation centers in California, Oregon, Washington and Alaska. (XLSX)

S2 Dataset Beach survey data on common murres summarized monthly, including survey effort, counts, and 95% CIs of mean murre encounter rate. (XLSX)
