# Supplementary material for: Extreme mortality and reproductive failure of common murres resulting from the northeast Pacific marine heatwave of 2014-2016
Source: PLoS One. 2020 Jan 15;15(1):e0226087. doi: 10.1371/journal.pone.0226087 (PMC6961838; doi:10.1371/journal.pone.0226087)
Supplement: S1 Table — (DOCX) [file pone.0226087.s001.docx]

**S1 Table. Wildlife rehabilitation organizations contacted in California, Oregon, Washington, British Columbia and Alaska.** Pertinent data on the recovery, treatment and disposition of common murres in their areas during the time (2015-2016) of the common murre die-off in the NE Pacific.

| **#** | **Name of Center** | **City** | **State/Prov** | **Nation** | **Reply?** | **Data** | **# Murres** |
| --- | --- | --- | --- | --- | --- | --- | --- |
| 1 | BC SPCA Wild Animal Rehabilitation Centre | Victoria | British Columbia | Canada | yes | yes | 11 |
| 2 | BC Wildlife Park Wildlife Rehabilitation Centre | Kamloops | British Columbia | Canada | yes | no | 0 |
| 3 | Doucette (Oiled Wildlife Society) |  | British Columbia | Canada | no | -- | -- |
| 4 | Elizabeth’s Wildlife Centre Society | Abbotsford | British Columbia | Canada | yes | no | 0 |
| 5 | Focus Wildlife Canada | Vancouver | British Columbia | Canada | yes | no | 0 |
| 6 | Gibsons Wildlife Rehabilitation Centre | Gibsons | British Columbia | Canada | yes | no | 0 |
| 7 | Mountainaire Avian Rescue Society | Courtenay | British Columbia | Canada | no | -- | -- |
| 8 | Northern Lights Wildlife Society | Smithers | British Columbia | Canada | yes | no | 0 |
| 9 | Owl Orphaned Wildlife | Delta | British Columbia | Canada | yes | no | 0 |
| 10 | Pacific Northwest Raptors | Duncan | British Columbia | Canada | yes | no | 0 |
| 11 | Prince Rupert Wildlife Rehabilitation Shelter | Prince Rupert | British Columbia | Canada | yes | yes | 3 |
| 12 | South Okanagan Rehabilitation Centre for Owls | Oliver | British Columbia | Canada | yes | no | 0 |
| 13 | Wildlife Rescue Association | Burnaby | British Columbia | Canada | yes | yes | 1 |
| 14 | Yukon Wildlife Preserve | Whitehorse | Yukon | Canada | yes | no | 0 |
| 15 | Alaska Raptor Center | Sitka | Alaska | USA | yes | yes | 6 |
| 16 | Alaska Sealife Center | Seward | Alaska | USA | yes | yes | 130 |
| 17 | Alaska WildBird Rehabilitation | Big Lake | Alaska | USA | yes | yes | 406 |
| 18 | Alaska Wildlife Conservation Center | Girdwood | Alaska | USA | yes | no | 0 |
| 19 | Bird Treatment and Learning Center | Anchorage | Alaska | USA | yes | yes | 414 |
| 20 | Juneau Raptor Center | Juneau | Alaska | USA | yes | yes | 2 |
| 21 | Bird Rescue Center | Santa Rosa | California | USA | yes | yes | 35 |
| 22 | California Wildlife Center | Malibu | California | USA | yes | yes | 33 |
| 23 | Humboldt Wildlife Care Center | Bayside | California | USA | yes | yes | 79 |
| 24 | International Bird Rescue | various | California | USA | yes | yes | 702 |
| 25 | Lindsay Wildlife Experience | Walnut Creek | California | USA | yes | no | 18 |
| 26 | Montery County SPCA | Montery | California | USA | yes | yes | 648 |
| 27 | Native Animal Rescue | Santa Cruz | California | USA | yes | yes | 465 |
| 28 | Ohlone Humane Society | Fremont | California | USA | yes | no | 0 |
| 29 | Pacific Wildlife Care | Morro Bay | California | USA | yes | yes | 596 |
| 30 | Peninsula Humane Society Burlingame | Burlingame | California | USA | yes | yes | 358 |
| 31 | Project Wildlife | San Diego | California | USA | yes | yes | 26 |
| 32 | San Francisco Animal Care and Control | San Francisco | California | USA | yes | yes | 98 |
| 33 | Santa Barbara Wildlife Care Network | Santa Barbara | California | USA | yes | yes | 188 |
| 34 | SeaWorld California | San Diego | California | USA | yes | yes | 10 |
| 35 | Sonoma County Wildlife Rescue | Petaluma | California | USA | yes | yes | 21 |
| 36 | Suisin Wildlife Center | Suisan City | California | USA | yes | no | 0 |
| 37 | Sulphur Creek Nature Center | Hayward | California | USA | yes | yes | 2 |
| 38 | The Fund for Animals Wildlife Center | Ramona | California | USA | yes | no | 0 |
| 39 | Wetlands & Wildlife Care Center | Huntington Beach | California | USA | yes | yes | 31 |
| 40 | Whale Rescue Team | Los Angeles County | California | USA | yes | no | 0 |
| 41 | WildCare Solutions | San Rafael | California | USA | yes | yes | 219 |
| 42 | Wildlife Care Association of Sacramento | McClellan Park | California | USA | yes | no | 1 |
| 43 | Wildlife Center of Silicon Valley | San Jose | California | USA | yes | yes | 2 |
| 44 | Wildlife Rehabilitation Center | Paicines | California | USA | no | no | 0 |
| 45 | Wildwings of California | San Dimas | California | USA | yes | no | 0 |
| 46 | Yggdrasil Urban Wildlife Rescue | San Francisco | California | USA | yes | no | 0 |
| 47 | Audubon Wildlife Care Center | Portland | Oregon | USA | yes | yes | 6 |
| 48 | Chintimini Wildlife Center | Corvallis | Oregon | USA | yes | yes | 93 |
| 49 | Rowena Wildlife Clinic | The Dalles | Oregon | USA | yes | no | 0 |
| 50 | Turtle Ridge Wildlife Center | Salem | Oregon | USA | yes | no | 0 |
| 51 | Wildlife Center of the North Coast | Astoria | Oregon | USA | yes | yes | 372 |
| 52 | Wildlife Images Rehabilitation and Education | Grants Pass | Oregon | USA | yes | yes | 1 |
| 53 | Willamette Wildlife Rehabilitation | Eugene | Oregon | USA | no | -- | -- |
| 54 | 2nd Chance Wildlife Care Center | Snohomish | Washington | USA | yes | no | 0 |
| 55 | Discovery Bay Wildbird Rescue | Port Townsend | Washington | USA | yes | yes | 8 |
| 56 | Featherhaven | Enumclaw | Washington | USA | yes | no | 0 |
| 57 | Fidalgo Animal Medical Center | Anacortes | Washington | USA | yes | no | 0 |
| 58 | For Heaven’s Sake | Olympia | Washington | USA | yes | no | 0 |
| 59 | Grays Harbor Veterinary Services | Montesano | Washington | USA | yes | no | 0 |
| 60 | Northwest Raptor Center | Sequim | Washington | USA | no | -- | -- |
| 61 | Ocean Beach Animal Hospital | Longview | Washington | USA | yes | no | 0 |
| 62 | PAWS Wildlife | Lynnwood | Washington | USA | yes | yes | 6 |
| 63 | Puget Sound WildCare | Kent | Washington | USA | yes | yes | 3 |
| 64 | Sarvey Wildlife Care Center | Arlington | Washington | USA | yes | yes | 3 |
| 65 | Seattle Aquarium | Seattle | Washington | USA | no | -- | -- |
| 66 | Useless Bay Animal Clinic | Freeland | Washington | USA | yes | no | 0 |
| 67 | West Sound Wildlife | Bainbridge Island | Washington | USA | yes | no | 0 |
| 68 | Whatcom Humane Soc. Wildl. Rehab. Ctr. | Everson | Washington | USA | yes | yes | 2 |
| 69 | Wildlife Care Clinic | Oak Harbor | Washington | USA | no | -- | -- |
| 70 | Willipa Veterinary Service | Raymond | Washington | USA | yes | no | 0 |
| 71 | Wolf Hollow Wildlife Rehabilitation Center | Friday Harbor | Washington | USA | yes | yes | 1 |
| 72 | Yelm Veterinary Hospital | Yelm | Washington | USA | yes | no | 0 |
