# Supplementary material for: Extreme mortality and reproductive failure of common murres resulting from the northeast Pacific marine heatwave of 2014-2016
Source: PLoS One. 2020 Jan 15;15(1):e0226087. doi: 10.1371/journal.pone.0226087 (PMC6961838; doi:10.1371/journal.pone.0226087)
Supplement: S2 Table — (DOCX) [file pone.0226087.s002.docx]

**S2 Table. Common murre collection details and measures of mass, sex, and age class.**

| Source of birds | Collection dates |  | Mass |  |  | Sex | |  |  | Age* | | Method |
| --- | --- | --- | --- | --- | --- | --- | --- | --- | --- | --- | --- | --- |
|  |  | n | mean | s.d | n | % male | % fem. | n | % juv | % sub | % adu |  |
| 1993 Alaska murre die-off | Jan-Apr 1993 | 116 | 666.0 | 92.4 | 82 | 50 | 50 | 56 | 0 | 46 | 54 | bursa |
| Gulf of Alaska colonies | May-Sep, 1988-1999 | 219 | 1054.0 | 94.3 | 221 | 60 | 40 | 94 | 0 | 8 | 92 | bursa, metrics |
| ASC necropsy, GOA 57°-62° N | Nov 2015 - Mar 2016 | 97 | 715.2 | 79.9 | 105 | 33 | 67 | 36 | 11 | 25 | 64 | bursa, metrics, plumage |
| NWHC necropsy, GOA, Bering Sea | Apr 2015 - Feb 2016 | 90 | 711.0 | 95.0 | 87 | 34 | 66 | 101 | 21 | 11 | 68 | bursa, metrics |
| S/SC California, 32°-37° N | May 2015 - Apr 2016 | 402 | 682.7 | 78.00 | n.d. | n.d. | n.d. | 742 | 34 | <1 | 66 | metrics, plumage |
| NC/N California, 37°-42° N | May 2015 - Apr 2016 | 157 | 675.6 | 72.00 | n.d. | n.d. | n.d. | 390 | 55 | <1 | 45 | metrics, plumage |
| Oregon/Washington, 42°-48° N | May 2015 - Apr 2016 | 44 | 639.4 | 67.90 | n.d. | n.d. | n.d. | 166 | 62 | 0 | 38 | metrics, plumage |
| * Age: juvenile=juv=HY, subadult=sub=ASY/ATY, adult=adu=AHY | | | |  |  |  |  |  |  |  |  |  |
