# Supplementary figures and images for: Extreme mortality and reproductive failure of common murres resulting from the northeast Pacific marine heatwave of 2014-2016
Source: PLoS One. 2020 Jan 15;15(1):e0226087. doi: 10.1371/journal.pone.0226087 (PMC6961838; doi:10.1371/journal.pone.0226087)

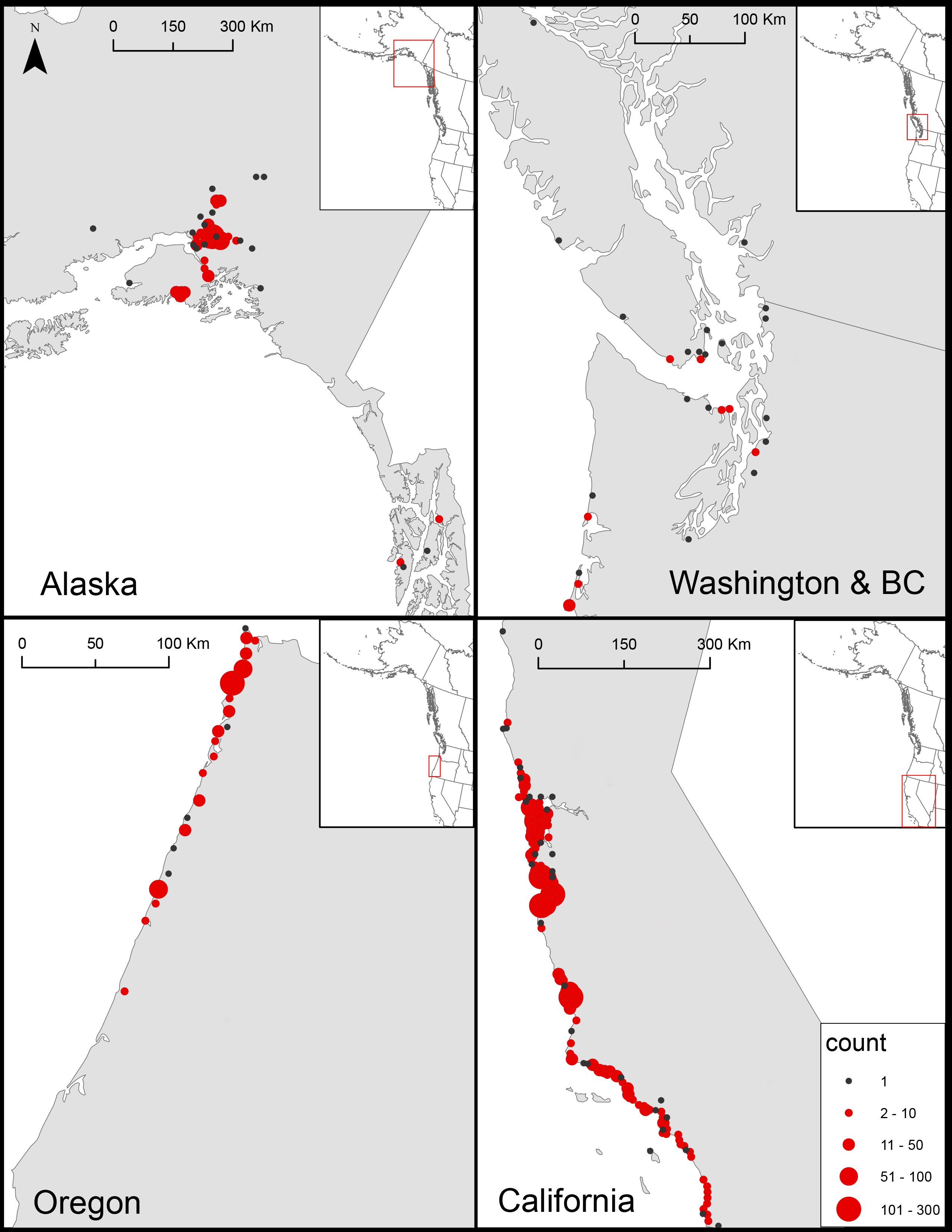

Supplement: S1 Fig — (TIF) [file pone.0226087.s003.tif]

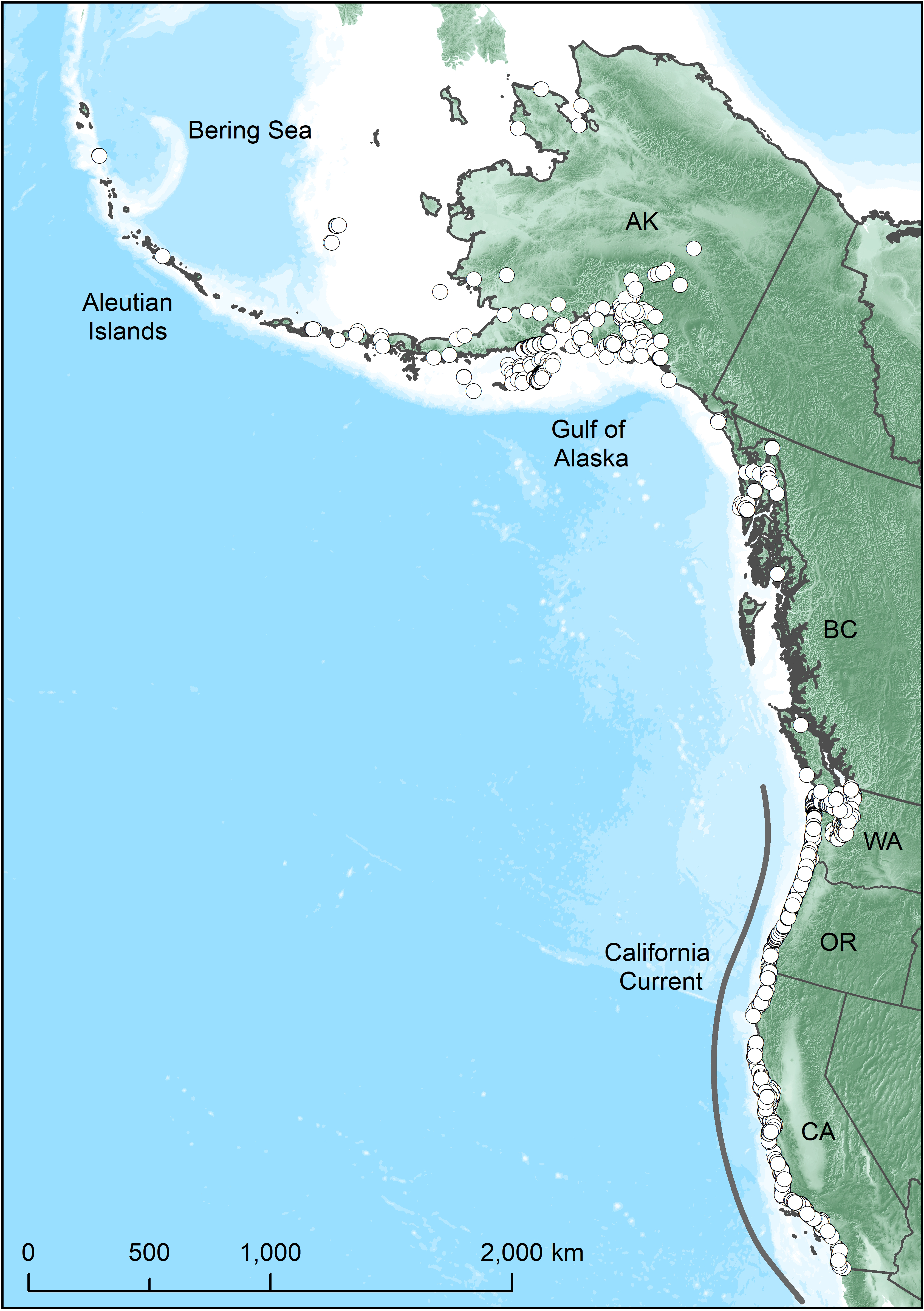

Supplement: S2 Fig — (TIF) [file pone.0226087.s004.tif]

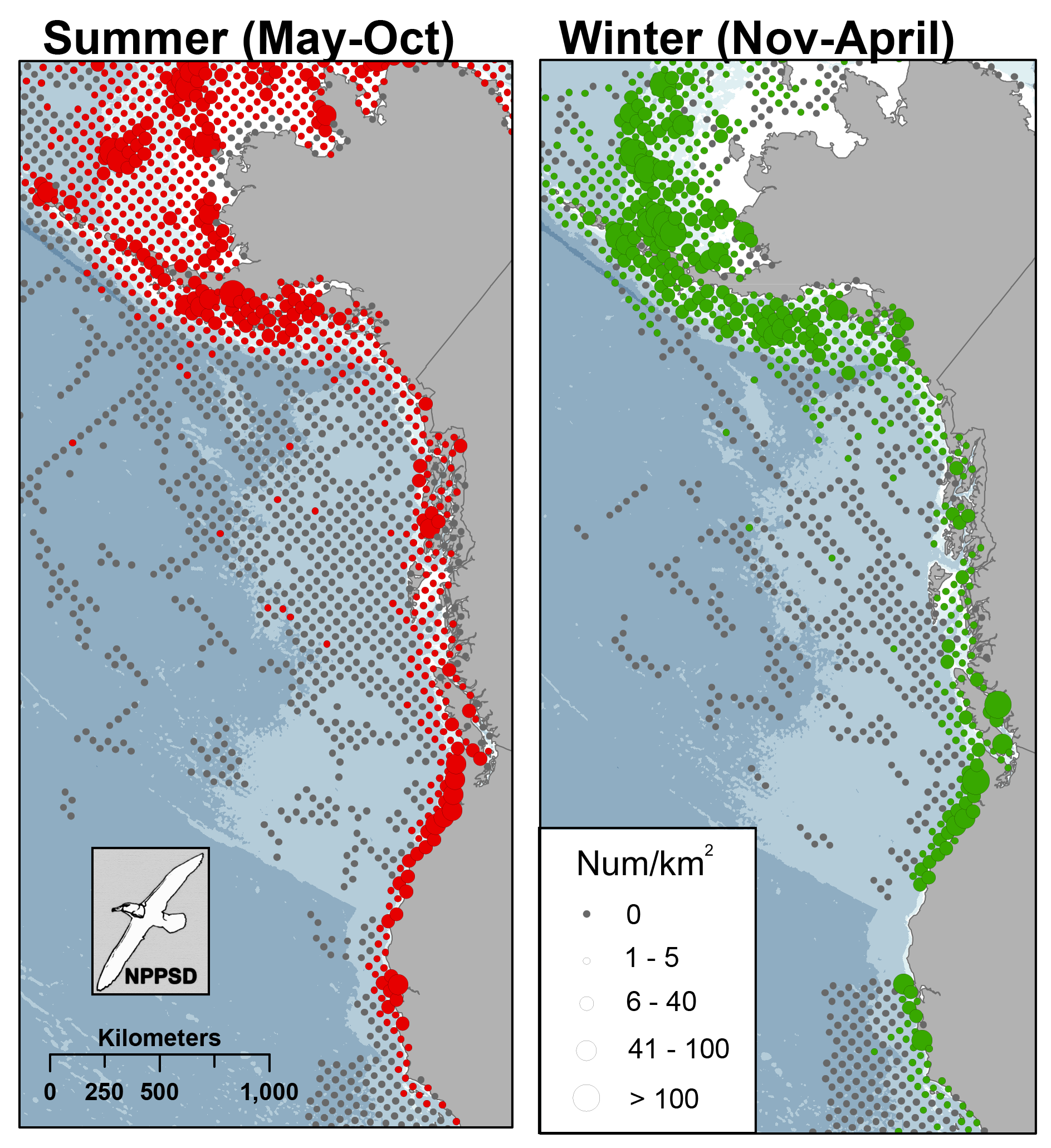

Supplement: S3 Fig — (TIF) [file pone.0226087.s005.tif]
