# Supplementary material for: Extreme mortality and reproductive failure of common murres resulting from the northeast Pacific marine heatwave of 2014-2016
Source: PLoS One. 2020 Jan 15;15(1):e0226087. doi: 10.1371/journal.pone.0226087 (PMC6961838; doi:10.1371/journal.pone.0226087)
Supplement: S1 Text — (DOCX) [file pone.0226087.s006.docx]

**S1 Text. Methods for bootstrap calculation of significance in testing rates of carcass encounter on beach surveys in different months and years.**

[supplementary material to Piatt et al. (2019) *Extreme mortality and reproductive failure of common murres resulting from the northeast Pacific marine heatwave of 2014-2016*; PLOS|ONE]

***Bootstrap significance***

***Methods***

In order to determine whether carcass encounter rates in 2015/16 were significantly higher than previous years we calculated bootstrap 95% confidence intervals of mean encounter rate at the region and month-year scale. Each bootstrap estimate was calculated by drawing n samples (with replacement) of survey-specific encounter rate from the pool of available surveys for that month-year and region (Gulf of Alaska, Outer coast of Washington, Oregon, N California, C California and SC California – see Table 1), with n equal to the number of unique beaches surveyed in that month-year. A distribution of mean encounter rate was then generated by performing 1,000 bootstrap permutations, subsequently processed to obtain a 95% confidence interval specific to that month-year and region.

The average for each calendar month (i.e. the baseline) was then calculated by a second round of bootstrap resampling using the distributions generated in the previous step. For each calendar month, n samples of month-year encounter rate were drawn from the distributions generated in the previous step, with n equal to the number of years surveyed prior to 2015 for that calendar month. Drawing from the distributions generated in the previous step allows for uncertainty in monthly encounter rate to be propagated through to the long-term average. A distribution of the mean for each calendar month and region were calculated by obtaining 1,000 bootstrap permutations of this procedure, which was subsequently converted to a mean and 95% confidence interval (Fig 1).


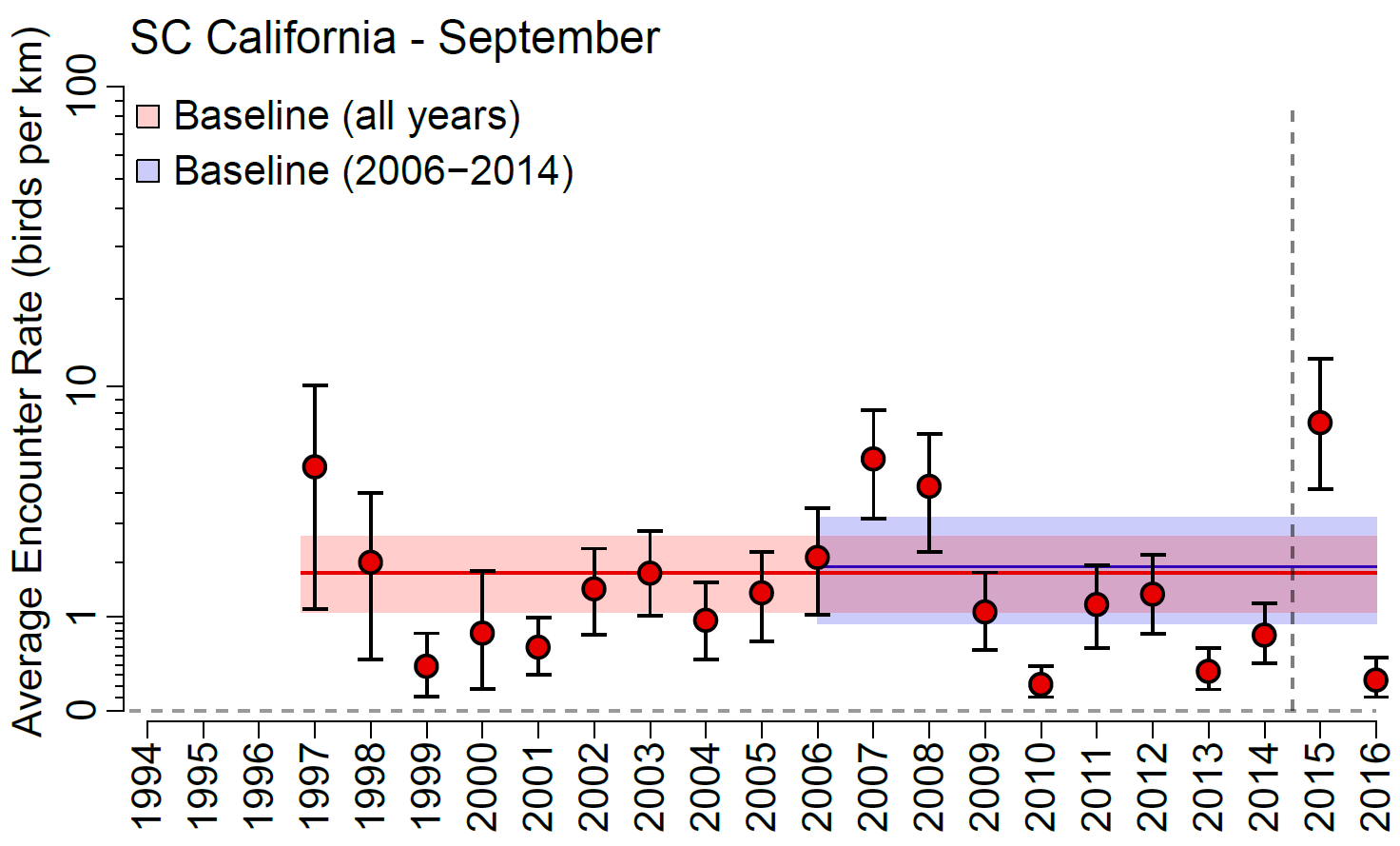


**Fig. 1. Month-averaged encounter rate of Murres recorded on surveys carried out in SC California in September plotted among years.** Each data point and bar represent a bootstrapped mean and 95% confidence interval calculated via resampling of survey-specific estimates within each month-year. The long-term average, or baseline, for this calendar month is shown as a mean and 95% confidence interval calculated via resampling of all years prior to 2014, and limited to surveyed years from 2006-2014.

Monthly encounter rates from May 2015 to April 2016 were then compared to the long-term baseline to identify whether they were significantly higher/lower than expected. We classified each month according to two significance criteria; (1) whether the encounter rate was significantly higher/lower than the long-term average (i.e. no overlap of corresponding 95% CI’s), (2) whether the encounter rate was significantly higher than any prior year for that calendar month (i.e. 2015/16 data was higher and had none overlapping 95% CI’s compared to all prior years). Whereas the first demonstrates that encounter rates were significantly higher than normal, the second identifies whether the corresponding encounter rate is higher/lower than previous maximums that may be associated with prior mortality events. In addition, we also recorded whether the corresponding mean encounter rate was the highest on record for that region and calendar month, irrespective of overlapping/non-overlapping 95% CI’s.

**Results**

In the Gulf of Alaska month-averaged encounter rates were the highest recorded (relative to monthly baselines: 2006-2014) from May 2015 through to March 2016, with the exception of June 2015, with the majority representing a statistically significant departure from baseline (Figure 2). In addition, from September 2015 to January 2016, month-averaged encounter rates were significantly higher than any previous year of data collection in the Gulf of Alaska (Figure 2). In Washington and Oregon, encounter rates were statistically higher than baseline from August to September of 2015, and represented the highest encounter rates ever recorded for those calendar months in Washington (Figure 2). Although encounter rates were high in Washington and Oregon, 95% CIs overlapped with several previous month-years (WA: Sep-2003, OR: Aug-2003, Aug-2004, Aug-2005, Aug-2009, Sep-2005) indicating that these months weren’t significantly higher than all previous years. In northern California encounter rates were at or below average, with encounter rates in November of 2015 being significantly lower than baseline (Figure 2). In central California, encounter rates were significantly higher than baseline and were the highest on record for September to November, and into December in South-central California (Figure 2). However, confidence intervals for these months overlapped with one or more prior years of data collection (N-C CA; Sep 1995, 2008, Oct 2006, Nov 1997, 2006, C-CA: Sep 1997, 2007, 2008, Oct 2008, Dec 2006) (Figure 2).


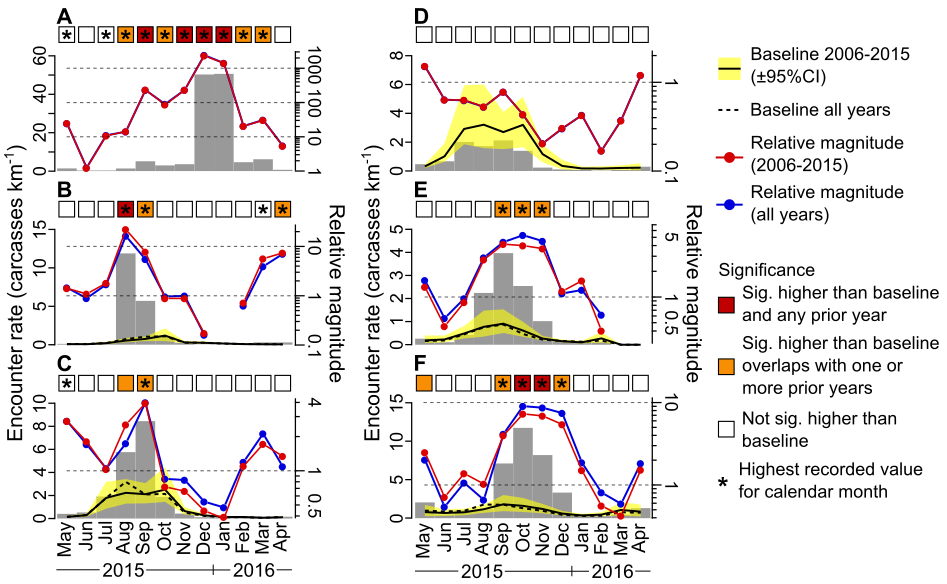


**Figure 2. Monthly averaged encounter rates (carcasses per km, gray bars) for the (A) Gulf of Alaska, (B) Washington, (C) Oregon, (D) Northern California, (E) North-Central California, and, (F) South-Central California coastlines**. Black lines are baseline encounter rates, yellow shadings are 95% confidence intervals, and colored lines show relative magnitude of encounter rates in 2015/2016 compared to baseline. Colored squares indicate whether month-averaged encounter rate was significantly higher than baseline (based on non-overlapping 95% CI’s relative to 2006-2014 baseline) and whether they were significantly higher than any prior year of data collection for that calendar month. Baselines and relative magnitudes are shown using “all years” of data in each area or using only those years (2006-2015) when data were collected in all areas at the same time. * in colored squares indicate that the mean encounter rate in the corresponding month was higher than all prior years of data collection (irrespective of 95% CI) for that calendar month and region. Note that the GOA baseline is so low that it cannot be seen on the chart. Relative magnitude was calculated as the 2015/2016 encounter rates divided by the baseline mean value.

Prior years that had comparable encounter rates (i.e. overlapping 95% confidence intervals of mean encounter rate include 2012 in the Gulf of Alaska, 2003-2005 and 2009 in Washington and Oregon, 1997-1998, 2005-2007 and 2008 in central California (Table 1).

**Table 1. Years with 95% confidence intervals of mean month-averaged Murre encounter rate (ER: birds per km) that were comparable (overlapping 95% CIs), or exceeded, values for 2015/16, by calendar month and region.**

|  | Region | | | | | |
| --- | --- | --- | --- | --- | --- | --- |
| Month | GoA | WA | OR | N CA | NC CA | C CA |
| May-15 | - | - | - | - | - | 1997^b^; 1998^a,b^;  2005^b^; 2006;  2007 |
| Jun-15 | - | - | - | - | - | - |
| Jul-15 | - | - | - | - | - | - |
| Aug-15 | 2012^a^; 2014 | ** | 2003; 2004^a,b^;  2005^b^; 2009 | - | - | - |
| Sep-15 | ** | 2003^a^ | 2005; 2008^a^ | - | 1995; 2008^a^ | 1997; 1998;  2007^a^; 2008 |
| Oct-15 | 2012^a^ | - | - | - | 2006^a^ | ** |
| Nov-15 | ** | - | - | - | 1997; 2006^a^ | ** |
| Dec-15 | ** | - | - | - | - | 2006^a^ |
| Jan-16 | ** | - | - | - | - | - |
| Feb-16 | 2012^a^ | - | - | - | - | - |
| Mar-16 | 2012^a^ | - | - | - | - | - |
| Apr-16 | - | 2002^a^; 2003;  2005; 2006;  2007; 2011 | - | - | - | - |

- : ER not significantly higher than baseline

^a^ : previous maximum

^b^ : mean ER exceeds mean ER for corresponding 2015/2016 month

** : 2015/2016 represents highest recorded value and is significantly higher than all prior years
